# Multiple-omics analysis of three novel haloalkaliphilic species of *Kocuria* revealed that the phenolic acid-degrading abilities are ubiquitous in the genus

**DOI:** 10.3389/fmicb.2025.1626161

**Published:** 2025-07-30

**Authors:** Lian Xu, Rui-Qi Sun, Jia-Hui Zeng, Hua-Mei Wei, Biao Shen, Ji-Quan Sun

**Affiliations:** ^1^Jiangsu Key Laboratory for Organic Solid Waste Utilization, Educational Ministry Engineering Center of Resource-saving Fertilizers, Jiangsu Collaborative Innovation Center for Solid Organic Waste Resource Utilization, Nanjing Agricultural University, Nanjing, China; ^2^Ministry of Education Key Laboratory of Ecology and Resource Use of the Mongolian Plateau, School of Ecology and Environment, Inner Mongolia University, Hohhot, China

**Keywords:** phenolic acids, biodegradation, *Kocuria*, comparative genomic analysis, osmotic stress, polyphasic taxonomy

## Abstract

Phenolic acids (PAs), which can exert toxic effects on seed germination and plant growth, are the most common allelopathic substances found in soils. To better understand the degradation fates of PAs in the rhizosphere of halophytes, five haloalkaliphilic PA-degrading bacteria, which were identified as three novel species of *Kocuria* (namely, *Kocuria rhizosphaerae* sp. nov., *Kocuria kalidii* sp. nov., and *Kocuria rhizosphaericola* sp. nov.), were obtained from the rhizosphere and bulk soil of the halophyte *Kalidium cuspidatum*. All five *Kocuria* strains could efficiently degrade ferulic acid (FA) and cinnamic acid (CA) under saline-alkaline conditions. Genomic and transcriptomic analyses revealed that the acrylic groups of FA and CA were first converted to a carboxyl via the coenzyme A (CoA)-dependent non-β-oxidation pathway by the five *Kocuria* strains. However, the five *Kocuria* strains selected different aromatic ring-cleavage ways for the degradation of the benzoic derivatives intermediates of the two compounds. The protocatechuate result from FA was then thoroughly degraded through an aromatic ring-opening reaction catalyzed by protocatechuate 3,4-dioxygenase (PcaGH), and the β-ketoadipic acid pathway. At the same time, the yield of benzoate originated from CA was subsequently converted to catechol by the benzoate 1,2-dioxygenase system (BenABCD) or phenylacetyl-CoA epoxidase (PaaABCD) and further completed the ring-cleavage by catechol 1,2-dioxygenase or catechol 2,3-dioxygenase (two non-PcaGH dioxygenases). The comparative genomic analysis revealed that the genes for phenolic acids hydroxylation, protocatechuate 3,4-dioxygenation, and those involved in the β-ketoadipic acid pathways are universal in the *Kocuria* strains. It is also demonstrated that the *Kocuria* strains maintain their osmotic balance by accumulating potassium, rather than biosynthesizing organic osmoprotectants, under hypersaline conditions.

## Highlights

- Five haloalkaliphilic phenolic acid (PA)-degrading bacteria isolated from the rhizosphere of halophyte.
- The five strains were identified as three novel species within the genus *Kocuria*.
- The *Kocuria* genus exhibits exceptional genetic versatility and adaptive capability.
- The five strains used different pathways to degrade ferulic acid (FA) and cinnamic acid (CA).
- Phenol acid-degrading abilities are universal in the genus *Kocuria*.

## 1 Introduction

Phenolic acids (PAs), as the most common allelochemicals in ecosystems, usually refer to the hydroxylated derivatives of benzoate and cinnamic acids (CA), that is, CA, ferulic acid (FA), *p*-hydroxybenzoate (PHA), protocatechuate (PCA), and vanillic acid (VA). These compounds are common intermediates during the decomposition of lignin and can also be secreted by the plant roots (Margesin et al., 2021). In addition, PAs are also common pollutants in industrial wastewater and sewage (González et al., 1990; Marchiosi et al., 2020). Majority of the PAs have been demonstrated to have antibiotic capacity. For example, FA not only irreversibly damaged the bacterial cell membrane but also significantly inhibited the adhesion of bacterial cells, the formation of biofilms, and the bacterial community structures (Borges et al., 2012; Kang et al., 2020; Matejczyk et al., 2024; Rao et al., 2023). A high concentration of PAs—a type of autotoxic substance in the soil—can inhibit the growth and development of plants, resulting in crop yield reduction (Ma et al., 2023; Xie et al., 2023).

Biodegradation by microbes is a crucial mechanism for regulating the concentration of PAs in the soil ecosystem. Many microbes from multiple habitats were found to be able to effectively metabolize various PAs in the soil environments (Margesin et al., 2021, 2022; Wang et al., 2022) and industrial process (Genethliou et al., 2020; Liu et al., 2022; Oshlag et al., 2020). However, fewer reports concerned the degradation fates of PAs in hypersaline-alkaline conditions, except two PAs-degrading bacteria from the rhizosphere soil of the halophyte *Suaeda salsa*, namely, *Acinetobacter suaedae* C16S1 and *Devosia rhizosphaerae* RR2S18 (Tian et al., 2024; Xu et al., 2024b).

CA and FA are the two most common PAs in rhizosphere soils. The degradation of the two PAs occurs mainly through two major strategies. The first strategy is to convert the acryl group to the carboxyl before the aromatic ring. For example, majority of bacteria initiate the degradation of the two compounds by converting them to central ring-fission intermediates [protocatechuic acid (PCA) or benzoic acids], catalyzed step by feruloyl-CoA synthase, hydroxycinnamoyl-CoA hydratase-lyase, and aldehyde dehydrogenase (Lubbers et al., 2019). The produced central intermediates are then consecutively degraded in a ring-cleavage step and via β-ketoadipic acid pathways to various low-molecular-weight organic acids, which finally enter the tricarboxylic acid (TCA) cycle (Harwood and Parales, 1996). Another strategy involved the two kinds of compounds undergoing aromatic ring fission without converting the acryl group to a carboxyl group. For example, the CA was directly oxidized into cinnamic acid-dihydrodiol and then to 2,3-dihydroxycinnamic acids through a set of enzymes encoded by the *hca* cluster. The produced 2,3-dihydroxycinnamic acids undergo extradiol ring cleavage and are ultimately degraded to Krebs cycle intermediates (Díaz et al., 1998).

**Figure 1:**
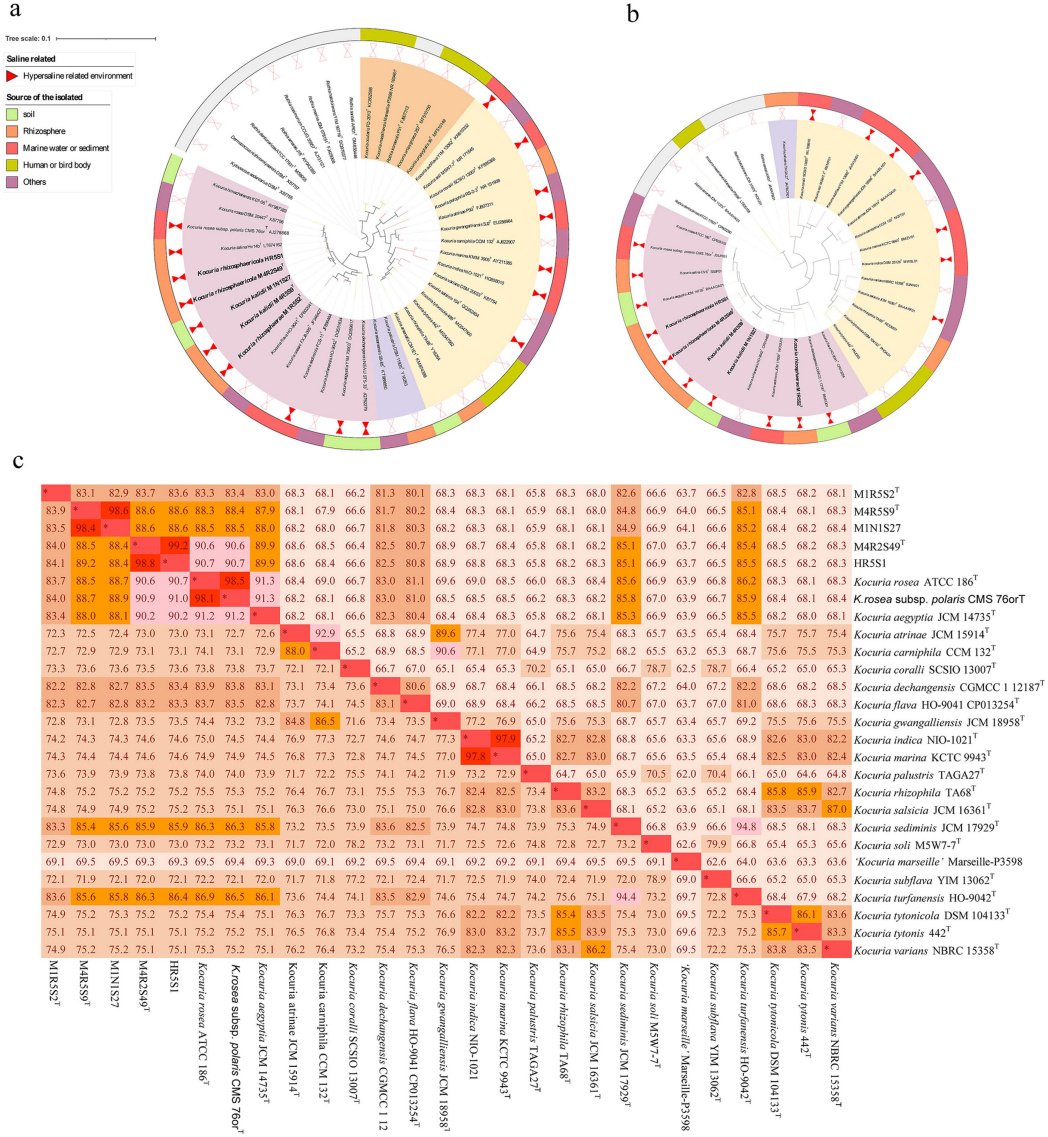
A phylogenetic tree based on the 16S rRNA gene sequences constructed using the neighbor-joining algorithm **(a)**, and a phylogenomic tree based on 876 core genes using OrthoFinder **(b)** Both bars 0.1 substitutions per nucleotide **(a)**/amino acids **(b)** position. The isolation source and clusters for strains were marked with different colors. The ANI and AAI values between the *Kocuria* strains **(c)**.

Members of the genus *Kocuria*, belonging to the family *Micrococcaceae* within the phylum *Actinomycetota*, are usually characterized as coccoid, strictly aerobic, Gram-stain positive, mesophilic bacteria (Stackebrandt et al., 1995). Majority of them are recognized as halophilic and sourced from high-salt related environments (Figure 1a). Some of them have been exhibited to be versatile at degrading pollutants, such as polycyclic aromatic hydrocarbons, naphthalene (Ezima et al., 2024; Cui et al., 2024; Khandelwal et al., 2024), and pentachlorophenol (Karn et al., 2011), along with heavy oil (Huang et al., 2012) and poultry feathers (Coello et al., 2000). This suggests that the members of *Kocuria* may play an important role in recycling carbon and energy in saline environments. Although a substantial amount of research has shown that the genus is present in hypersaline habitats, there has been less research on the ecological roles of the genus in the environment.

During an ongoing study on the plant-bacteria interaction, a total of 2,670 isolates were collected from the rhizosphere soil and non-rhizosphere soil of *Kalidium cuspidatum*, a common halophyte in the northwestern inner continent of China. Among them, five PAs-degrading strains of *Kocuria* shared low 16S ribosomal RNA (rRNA) gene similarities with current strains, and exhibited high PA-degrading abilities under saline-alkaline conditions. To better understand the roles of the genus in PAs degradation, we will elucidate their accurate taxonomic position and their PAs-degrading mechanism through multiple-omics analyses.

## 2 Materials and methods

### 2.1 Source of the strains

The soil samples used to isolate the strains were collected from the rhizosphere of *K. cuspidatum* and the bulk saline soil in Tumd Right Banner, Baotou, Inner Mongolia, China. Approximately 1.0 g rhizosphere soil was collected by hand-shaking roots to remove the adhering soil (Barillot et al., 2013), while the bulk soil was collected from soil without any plants growing next to the plants. Among them, two samples of rhizosphere soil were designated R2 and R5, while the bulk saline soil sample was designated N1. The values of the pH, concentration of salt, and organic matter were 9.1, 3.20 mS cm^−1^, and 2.94 g kg^−1^ for R2, 10.2, 6.63 mS cm^−1^, and 1.09 g kg^−1^ for R5, and 9.3, 7.77 mS cm^−1^, and 2.9 g kg^−1^ for N1 (Xu et al., 2024a), respectively. The strains were isolated using a protocol as previously described. Briefly, the 10-fold diluted soil suspensions were plated on M1 agar (g L^−1^: 2 sodium pyruvate, 1.0 L-asparagine, 0.1 (NH_4_)_2_SO_4_, 0.1 KCl, 30 MgSO_4_·7H_2_O, 0.05 FeSO_4_·7H_2_O, and 20 agar), M4 agar (g L^−1^: 2.5 cellulose, 2.0 sodium pyruvate, 0.25 KNO_3_, 1.0 proline, 0.2 MgSO_4_·7H_2_O, 0.2 K_2_HPO_4_, 0.5 CaCl_2_, 0.01 FeSO_4_·7H_2_O, 30 NaCl, and 20 agar), and artificial seawater agar (g L^−1^: 24.0 NaCl, 5.4 MgCl_2_·H_2_O, 5.0 tryptone, 4.0 Na_2_SO_4_, 1.5 CaCl_2_·H_2_O, 1.0 yeast extract, 0.68 KCl, 0.5 NH_4_Cl, 0.1 KBr, 0.2 NaHCO_3_, 0.2 Na_2_HPO_4_, 0.025 H_3_BO_3_, 0.024 SrCl_2_·6H_2_O, 0.002 NaF, and 20.0 agar, pH 8.0). The inoculated plates were then incubated at 30°C in the dark for a week. The colonies present on the agar plates with typical characteristics were picked and purified on new agar plates. The purified isolates were deposited in 15% glycerol solutions at −80°C. The isolates’ PAs-degrading capacities were primarily determined according to their growth in modified minimal salt medium (MMSM; g L^−1^: 3.5 K_2_HPO_4_, 0.2 KH_2_PO_4_, 30 NaCl, 1.0 (NH_4_)_2_SO_4_, and 0.2 MgSO_4_ 7H_2_O; pH 8.0) which was added 100 mg L^−1^ PHA as sole organic carbon source using a protocol as previously described (Xu et al., 2024b).

Strain *Kocuria rosea* CGMCC 4.7049^T^ (= ATCC 186^T^), which was used as a reference for the chemotaxonomic and phenotypic tests, was obtained from the China General Microbiological Culture Collection Center (CGMCC).

### 2.2 Phylogenetic analysis based on the 16S rRNA gene and whole genome

After 2 days of cultivation in high-salt LB broth (LB: g L^−1^: 5.0 yeast extract, 10.0 tryptone, and 30 NaCl; pH 8.0), the collected cell pellets were used as sources to for extract the total DNA using a DNA extraction kit (TianGen, Beijing, China) following the manufactures’ specification. The 16S rRNA genes of the five strains were amplified using universal primer sets (27F/1492R) and sequenced as previously described (Sun et al., 2017). The almost complete 16S rRNA gene sequences obtained were first used to determine their primary taxonomic positions using Basic Local Alignment Search Tool (BLAST) in NCBI.^1^ Their accurate taxonomic positions were determined by constructing a phylogenetic tree of the five strains along with the reference strains using the neighbor-joining (NJ) algorithm in MEGA software version 6.1 (Tamura et al., 2013). The topology of the tree was evaluated using the bootstrap methods.

To sequence the whole genome of the five strains, the short-insert (~350 bp) libraries were constructed using the Illumina library preparation kit (Illumina, San Diego, CA, United States) according to the manufacturer’s instructions. All the libraries were then sequenced using an Illumina NovaSeq platform with the paired-end 150 (PE150) strategy. The raw reads were subsequently trimmed for quality using Trimmomatic version 0.35. The clean data were used for subsequent analyses. The draft genomes were assembled using SOAPdenovo2. Contig statistics, including N50 and the largest contig size, were calculated using QUality ASsessment Tool (QUAST) (Gurevich et al., 2013). The depth of coverage was determined using BEDTools (Quinlan and Hall, 2010). The sequencing generated 332–575-fold mean coverage for the genomes of the five strains. The authenticity of the genomes was checked using 16S rRNA genes. The completeness and contamination of the whole genome of the five strains were evaluated using CheckM (Parks et al., 2015). The completeness and contamination values of the strains and the reference strains are provided in Supplementary Table S1. The overall genome indices, including calculation of average nucleotide identity b (ANIb) and average amino-acid identity (AAI) values, were analyzed using fast alignment-free computation of whole-genome average nucleotide identity (FastANI) (Hernández-Salmerón and Moreno-Hagelsieb, 2022) and EzAAI (Kim et al., 2021), respectively. A phylogenomic tree was constructed based on 876 core genes using OrthoFinder (Emms and Kelly, 2015, 2019) with default parameters.

### 2.3 Polyphasic taxonomy

The growth range of temperature, pH, and concentration of NaCl of the five strains was determined in a modified LB broth as previously described (Xu et al., 2024a). The abilities to utilize carbon were determined in MMSM as previously described (Xu et al., 2022). The cell morphology was observed using a transmission electron microscope (HT7800; Hitachi, Tokyo, Japan).

The polar lipids of these five strains, along with their common reference strain *K. rosea* CGMCC 4.7049^T^, were extracted using a chloroform and methanol (1:2; v/v) mixture and detected using thin-layer chromatography as previously described (Kates, 1986). The respiratory quinones of these strains were extracted with chloroform/methanol (2:1; v/v), and determined using a high-pressure liquid chromatograph (HPLC; LC-16A; Shimadzu) equipped with a photodiode array detector (SPD-M40A) and a Kromasil 100^−5^ C18 separation column (internal diameter, 4.6 mm; length, 20 cm) as previously described (Komagata and Suzuki, 1987).

### 2.4 Degradation of the PAs

The PA-degrading abilities of the five strains were determined in MMSM with a protocol described in the literature (Tian et al., 2024). The inocula of the five strains were prepared in LB broth and harvested by centrifugation at 3,000*g* for 5 min. The cell pellets were rinsed and resuspended in MMSM at a final concentration of optical density (OD_600_) of 1.0. The cell suspensions were subsequently inoculated at 1% (v/v) into MMSM that contained 150 mg L^−1^ FA or 150 mg L^−1^ CA. The inoculated cultures were incubated at 150 r min^−1^ and 30°C in the dark. MMSM that contained PAs but were not inoculated with the strains were used as a control to evaluate the natural loss of the compounds. The cultures were sampled at 12, 24, 36, 48, 72, 96, 156, 204, 252, 300, 348, and 396 h.

The cell densities in the cultures were determined using an ultraviolet (UV) spectrophotometer (Shimadzu UV-1780, Shimadzu, Kyoto, Japan). The concentration of the residual PAs was determined using an HPLC equipped with a photodiode array detector (SPD-M40A) and a Kromasil 100^−5^ C18 separation column following the protocol ass previously described (Sun et al., 2012). Methanol:water (7:3, v/v) was used as the mobile phase at a flow rate of 0.7 mL min^−1^. FA and CA were recorded at 287 and 288 nm and 30°C with the detection limit being 0.01 mg L^−1^.

### 2.5 Transcriptomic analyses

Strains M4R2S49^T^, M1R5S2^T^, and M4R5S9^T^ were selected for the transcriptomic analyses. Cells of the three strains in MMSM that contained CA or FA were harvested at 9 h of incubation for the total transcriptome analysis. The total RNA was extracted and evaluated as previously described (Xu et al., 2024b).

A total of 3 μg of RNA per sample was used to construct the sequencing libraries with an NEBNext® Ultra™ Directional RNA Library Prep Kit for Illumina® (NEB, Ipswich, MA, United States) according to the manufacturer’s instructions. The rRNA removal, RNA fragmentation, first-strand cDNA synthesis, second-strand cDNA synthesis, conversion of the remaining overhangs to blunt ends, and adenylation of 3′ ends of DNA fragments were performed as previously described (Xu et al., 2024b). The library fragments were then purified using an AMPure XP system (Beckman Coulter, Brea, CA, United States) to select cDNA fragments of 150–200 bp in length. After cluster generation on a cBot Cluster Generation System using a TruSeq PE Cluster Kit v3-cBot-HS (Illumina), the library preparations were sequenced on an Illumina HiSeq platform, and paired-end reads were generated. The transcriptomic data were first normalized using glyceraldehyde-3-phosphate dehydrogenase (GAPDH) gene as the reference, and taking the sample cultivated with tryptone as the control.

### 2.6 Comparative genomic analyses

As of July 2024, 232 genomes of the *Kocuria* strain, including metagenome-assembled genomes (MAGs) deposited in GenBank. For the accuracy of the analysis, only 168 genomes with high quality (completeness >99% and contamination <1%) were selected for comparative genomic analysis. The pan- and core-genomes of the *Kocuria* strains were analyzed using the Bacterial Pan Genome Analysis tool (BPGA) pipeline version 1.3 (Chaudhari et al., 2016). The pan-genome genes were sorted out using Roray (Brynildsrud et al., 2016) with the default parameters.

### 2.7 Deposition of data

The 16S rRNA and whole genome sequence for these five strains were deposited in GenBank/EMBL/DDBJ under the accession numbers PQ498817-PQ498821, and JBISWJ000000000, JBISWK000000000, JBISWL000000000, JBISWM000000000, and JBISWN0000000000, respectively.

## 3 Results

### 3.1 Isolation and taxonomy of the strains

A total of five haloalkaliphilic strains, which were isolated from the rhizosphere soil of *K. cuspidatum* (namely strains HR5S1, M1R5S2^T^, M4R5S9^T^, and M4R2S49^T^) and bulk saline soils (strain M1N1S27), were selected for this study. A phylogenetic tree based on the 16S rRNA genes showed that the five strains clustered with *K. rosea* ATCC 186^T^, *K. rosea* subsp. *polaris* CMS 76or^T^, and *Kocuria himachalensis* K07-05^T^, and formed three independent clades: M4R5S9^T^ with M1N1S27, M4R2S49^T^ with HR5S1, and M1R5S2^T^ alone (Figure 1a). The five strains shared the highest 16S rRNA gene similarities with their partner within the clades and then with *K. rosea* ATCC 186^T^. However, the phylogenomic tree based on the core genome showed a somewhat different phylogeny relationship. For example, on the phylogenomic tree, only the clade of strains M4R2S49^T^ and HR5S1 tightly clustered with strains of *K. rosea* ATCC 186^T^ and *K. rosea* subsp. *polaris* CMS 76or^T^, and *K. himachalensis* K07-05^T^, while the two other clades were located outside of the branch (Figure 1b). The ANIb and AAI values between the strains within the two clades of M4R5S9^T^ and M1N1S27, and M4R2S49^T^ and HR5S1 were all >95.0%, indicating that the strains within each clade belonged to the same species (Chun et al., 2018). Meanwhile, all the ANIb and AAI values of the five strains from the other type strains, including their closest relative *K. rosea* ATCC 186^T^, were <95.0% (Figure 1c), demonstrating they can be differentiated from the current species. Many characteristics can be used to distinguish the five strains of three clades from their closest related strain, *K. rosea* ATCC 186^T^ (=CGMCC 4.7049^T^) (Supplementary Table S2). Based on the phylogenetic and phenotypic results, three clades formed by five new isolates should be identified as three novel species within the genus *Kocuria*. Therefore, *K. rhizosphaerae* sp. nov. (type strain: M1R5S2^T^ = CGMCC 1.64778^T^ = JCM 37379^T^), *Kocuria kalidii* sp. nov. (type strain: M4R5S9^T^ = CGMCC 1.64776^T^ = JCM 37381^T^; non-type strain: M1N1S27), and *Kocuria rhizosphaericola* sp. nov. (type strain: M4R2S49^T^ = CGMCC 1.64777^T^ = JCM 37380^T^; non-type strain: HR5S1) were proposed. The descriptions of the three novel species are shown in Table 1.

**Table 1:** Description of the three novel species within the genus *Kocuria*.

Genus name	*Kocuria*	*Kocuria*	*Kocuria*
Species name	*Kocuria rhizosphaerae*	*Kocuria kalidii*	*Kocuria rhizosphaericola*
Specific epithet	*rhizosphaerae*	*kalidii*	*rhizosphaericola*
Species status	sp. nov.	sp. nov.	sp. nov.
Species etymology	rhi. zo. sphae′rae. Gr. fem. n. *rhiza* a root; L. fem. n. *sphaera* a ball, sphere; N.L. gen. n. *rhizosphaerae* of the rhizosphere	ka.li’di.i. N. L. gen. n. *kalidii* of the plant *K. cuspidatum*	rhi.zo.sphae.ri’co.la. N.L. fem. n. *rhizosphaera*, the rhizosphere; L. masc./fem. n. suff. *-cola*, inhabitant, dweller; from L. masc./fem. n. *incola*, dweller; N.L. masc./fem. n. *rhizosphaericola*, inhabiting the rhizosphere
Description of the new taxon and diagnostic traits	Colonies on LB agar are pale yellow, circular, raised, and smooth. Cells are Gram-stain-positive, facultatively aerobic, non-spore-forming, non-motile, spherical (0.9–1.1 μm). Grows at a temperature range of 10–40°C (optimal 30–35°C), pH 6.0–11.0 (optimal pH 8.0), and in the presence of 0–20% NaCl (optimal 0–3%). Assimilates D-glucose, mannitol, D-maltose, gluconate, malic acid, and phenylacetic acid. Does not assimilate capric acid, trisodium citrate as sole carbon source for growth. Positive for nitrate reduction, nitrite reduction, arginine dihydrolase, urease, aesculin hydrolysis, β-galactosidase, esterase (C4), esterase lipase (C8), leucine arylamidase, valine arylamidase, naphthol-AS-BI-phosphohydrolase, β-glucuronidase, *α*-glucosidase, and β-glucosidase. Negative for indole production, glucose fermentation, alkaline phosphatase, lipase (C14), trypsin, α-chymotrypsin, acid phosphatase, α-galactosidase, *N*-acetyl-β-glucosaminidase, and *α*-fucosidase. Cystine arylamidase, α-mannosidase, and assimilation of L-arabinose, D-mannose, and adipic acid are variable. The major polar lipids are diphosphatidylglycerol (DPG), phosphatidylglycerol (PG), and an unidentified lipid (L). The major quinone is MK-7(H2).	Colonies on LB agar are orange, circular, raised, and smooth. Cells are Gram-stain-positive, facultatively aerobic, non-spore-forming, non-motile, spherical (1.4–1.5 μm). Grows at a temperature range of 10–40°C (optimal 30°C), pH 6.0–11.0 (optimal pH 9.0), and in the presence of 0–20% NaCl (optimal 5%). Assimilates D-mannose, mannitol, D-maltose, and gluconate; but does not assimilate *N*-acetyl-glucosamine and capric acid. Assimilation of D-glucose, L-arabinose, adipic acid, malic acid, trisodium citrate, and phenylacetic acid is variable. Positive for nitrate reduction, nitrite reduction, arginine dihydrolase, urease, aesculin hydrolysis, esterase (C4), esterase lipase (C8), lipase (C14), leucine arylamidase, valine arylamidase, naphthol-AS-BI-phosphohydrolase, β-glucuronidase, α-glucosidase, and β-glucosidase. Negative for indole production, glucose fermentation, gelatin hydrolysis, β-galactosidase, alkaline phosphatase, α-chymotrypsin, α-galactosidase, N-acetyl-β-glucosaminidase, α-mannosidase, and α-fucosidase. Cystine arylamidase, trypsin, and acid phosphatase are variable. The major polar lipids are diphosphatidylglycerol (DPG), and two unidentified lipids (L). The major quinone is MK-7(H2).	Colonies on LB agar are orange, circular, raised, and smooth. Cells are Gram-stain-positive, facultatively aerobic, non-spore-forming, non-motile, spherical (0.8–1.9 μm). Grows at a temperature range of 10–40°C (optimal 30°C), pH 6.0–11.0 (optimal pH 8.0), and in the presence of 0–10% NaCl (optimal 0%). Assimilates D-glucose, L-arabinose, mannitol, D-maltose, gluconate, adipic acid, malic acid; but does not assimilate *N*-acetyl-glucosamine and capric acid. Assimilation of D-mannose, trisodium citrate, and phenylacetic acid is variable. Positive for arginine dihydrolase, urease, esterase (C4), esterase lipase (C8), leucine arylamidase, valine arylamidase, cystine arylamidase, trypsin, naphthol-AS-BI-phosphohydrolase, α-glucosidase, and β-glucosidase. Negative for indole production, glucose fermentation, gelatin hydrolysis, β-galactosidase, α-chymotrypsin, α-galactosidase, β-glucuronidase, α-mannosidase, and α-fucosidase. Alkaline phosphatase, lipase (C14), acid phosphatase, and *N*-acetyl-β-glucosaminidase are variable. The major polar lipids are diphosphatidylglycerol (DPG), phosphatidylglycerol (PG), and an unidentified lipid (L). Major quinone is MK-7(H2).
Country of origin	China	China	China
Region of origin	Inner Mongolia: Baotou	Inner Mongolia: Baotou	Inner Mongolia: Baotou
Date of isolation	09/2018	09/2018	09/2018
Source of isolation	Rhizosphere soil	Rhizosphere soil	Rhizosphere soil
Sampling date	09/2018	09/2018	09/2018
Latitude	40°33′38″N	40°33′38″N	40°33′38″N
Longitude	110°46′54″E	110°46′54″E	110°46′54″E
Altitude (meters above sea level)	1,009 m	1,009 m	1,009 m
16S rRNA gene accession number	PQ498819	PQ498821	PQ498820
Genome accession number	JBISWL000000000	JBISWJ000000000	JBISWK000000000
Genome status	Draft genome sequence	Draft genome sequence	Draft genome sequence
Genome size	3,997,752 bp	3,757,048 bp	4,203,210 bp
GC %	72.4	72.6	72.0
Number of strains in study	1	2	2
Source of isolation of non-type strains		Bulk saline soil	Rhizosphere soil of *K. cuspidatum*
Designation of the type strain	M1R5S2^T^	M4R5S9^T^	M4R2S49^T^
Strain collection numbers	CGMCC 1.64778^T^; JCM 37379^T^	CGMCC 1.64776^T^; JCM 37381^T^	CGMCC 1.64777^T^; JCM 37380^T^
Additional strains of the species		M1N1S27	HR5S1

### 3.2 Characteristics of the degradation of PAs by the five *Kocuria* strains

**Figure 2:**
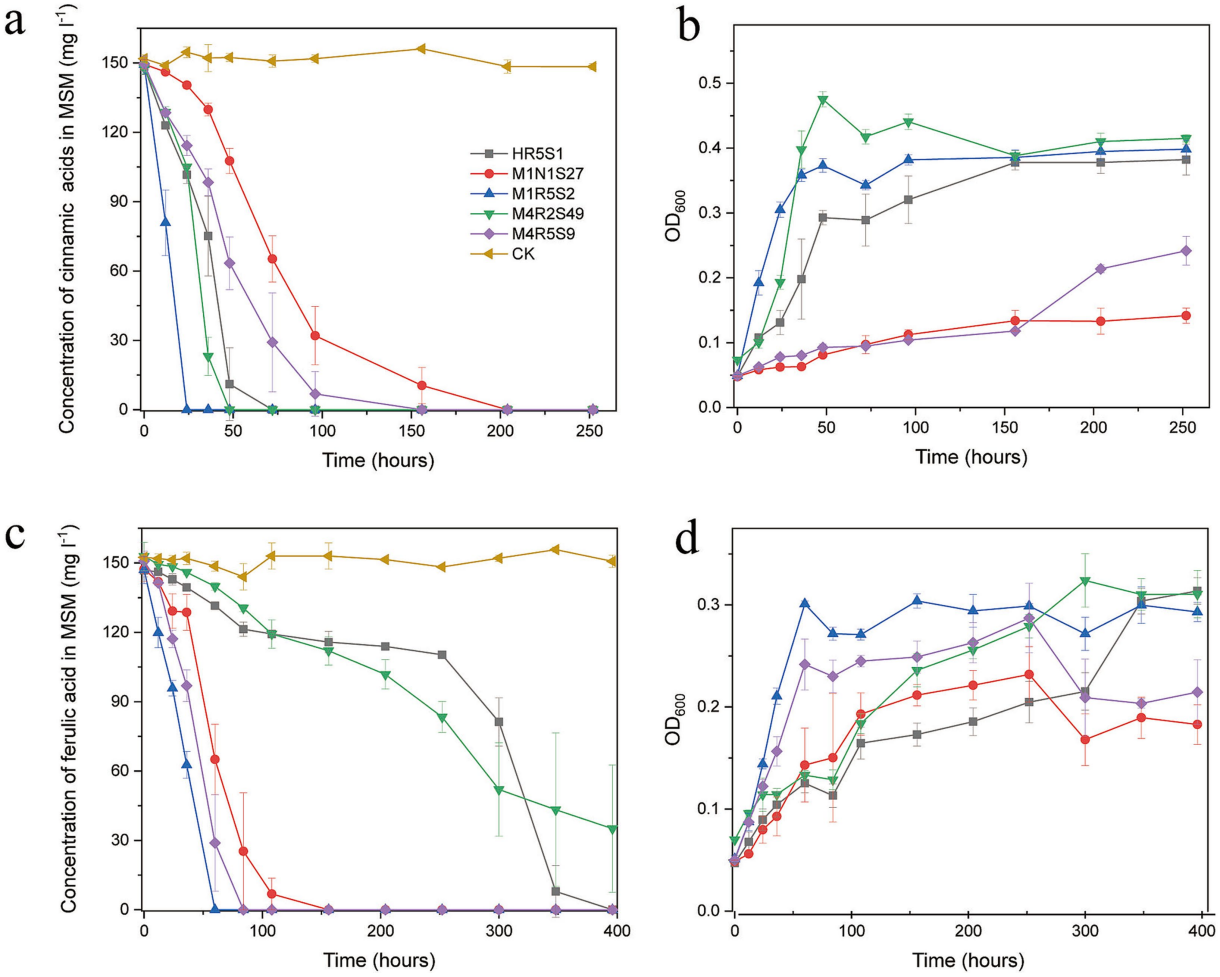
**(A,C)** The five *Kocuria* strains degraded FA and CA in MMSM (pH 8.0 and 3.0% NaCl) as the sole carbon and energy source for the strains’ growth. The initial concentration of both PAs is 150 mg L^−1^. **(B,D)** The biomass of the strains was determined by measuring the optical density at 600 nm (OD_600_)_._ The values in the figures are the average of the 3 times, while the bars are error bars. MMSM, which contained FA or CA but without inoculating any strains, was used as a control (CK) to evaluate the natural loss of compounds. All five strains did not obviously grow in MMSMs without any PAs (data not shown).

All five *Kocuria* strains efficiently degraded FA and CA in the presence of 3% NaCl at pH 8.0 (Figure 2). The strains belong to a species that exhibited similar degradation characteristics. Overall, the strains degraded CA quickly compared to FA, which may be attributed to the different molecular structures of these compounds. The presence of a methoxy group in FA made it difficult to degrade. In detail, strain M1R5S2^T^ degraded PAs the quickest: it completely degraded 150 mg L^−1^ CA within 24 h, and 150 mg L^−1^ FA within 60 h. While strains M4R5S9^T^ and M1N1S27 degraded the same amount of FA within 160 h, and CA within 210 h. Compared to strains M4R5S9^T^ and M1N1S27, strains M4R2S49^T^ and HR5S1 took much longer time (>300 h) to degrade the same amount of FA, but less time for the degradation of CA. Additionally, the degradation of PAs consistently increased the cell density. In detail, the cell densities (OD_600_) of the five strains increased from 0.05 to 0.1–0.45 in the samples that contained 150 mg L^−1^ FA as the sole carbon source, while the cell densities increased from 0.05 to 0.20–0.30 in the samples that contained 150 mg L^−1^ CA, indicating that these five strains could utilize these two kinds of PAs as the sole carbon source for strains’ growth (Figure 2). It is notable that the same amount of CA resulted in much less biomass from M4R5S9^T^ and M1N1S27 (OD_600_ < 0.2) compared to the three other strains, which corresponded to the lower degradation rate of CA by these two strains.

An intermediate metabolite, which shared a similar ultraviolet spectrum and retention time (5.6 min) to VA, was detected during the degradation of FA by strain M4R5S9^T^. The same metabolites were also detected during the degradation of FA by strains M1R5S2^T^ and M1N1S27 (data not shown).

### 3.3 Predicted degradation pathways in the five strains of *Kocuria*

**Figure 3:**
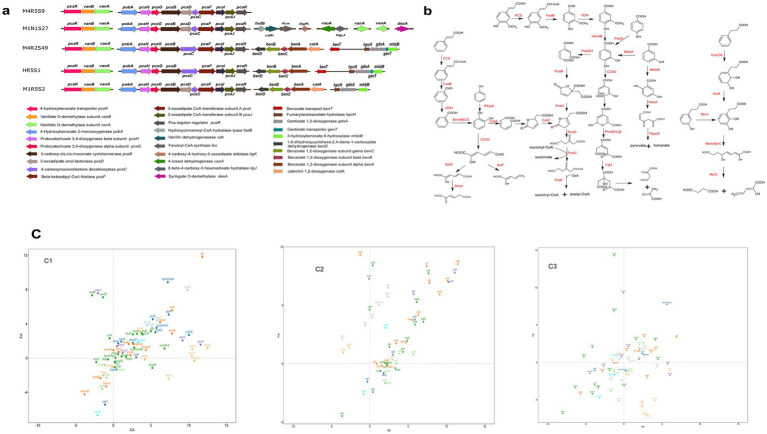
Genes related to aromatic compounds degradation in the genomes of the five strains **(a)**, and the predicted degradation pathways according to the annotated genes **(b)**, and the regulation of aromatic compounds-degrading genes in strains M1R5S2^T^ **(C1)**, M4R5S9^T^ (**C2**) and M4R2S49^T^ **(C3)** by FA and CA. The values of the genes were first normalized using the GDPAH gene as a reference and using cells cultivated in LB as the control. The normalized values were then used for logarithmic calculation using 1.5 as the base number. The *X*-axis represents the expression of the genes induced by CA, while the *Y*-axis reflects the expression of the related genes induced by FA. The values are positive for the expression of the genes. A value >0 means gene upregulation, while a value <0 means gene downregulation **(c)**. More detailed gene names are listed in Supplementary Table S3.

The CoA-dependent β-oxidation pathway, by which FA is converted to PCA, is a common way to degrade FA. This pathway is usually catalyzed by FCS, FadB, Vdh, and VanAB (Figure 3a). Although all five strains were proven to be capable of degrading CA and FA, only strain M1N1S27 was found to harbor a specialized feruloyl CoA synthesis gene (*fcs*) and hydroxycinnamoyl-CoA hydratase-lyase gene (*fadB*) (Figure 3a). However, there are many predicted enzymes with similar catalytic functions in the five strains. For example, the 2-succinylbenzoate-CoA ligase gene *menE* functions similarly to *fcs*, and an enoyl-CoA hydratase gene (*ech*) is similar in function to *fadB*. The transcriptomic analysis showed that *menE* and at least one of the enoyl-CoA hydratase genes were significantly upregulated by both PAs (Figure 3c). The annotated vanillin dehydrogenase was not upregulated. Still, a phenylacetaldehyde dehydrogenase (named *feaB*) was remarkably upregulated by both PAs. All five strains harbored the vanillate *O*-demethylase genes *vanAB*, which were responsible for the conversion of a methoxy group on the aromatic ring of vanillate (or its derivative) to a hydroxyl group. Because CA lacks a methoxy group, the vanillate o-demethylase genes *vanAB* should be upregulated by FA, and not upregulated by CA. The transcriptome result confirmed this hypothesis. There are three other *O*-demethylase genes (two vanillate *O*-demethylase genes and a syringate *O*-demethylase gene *desA*) in the genome of strain M1N1S27 (Figure 3a). However, all these demethylase genes were not upregulated by FA or CA, which suggests that the other O-demethylase genes may not be involved in the degradation of FA. The transcriptomic results indicated that all five strains could convert FA to PCA, and convert CA to benzoate via CoA-dependent β-oxidation pathways.

After the conversion of an acryl group to a carboxyl group on the aromatic ring of CA/FA by the CoA-dependent non-β-oxidation pathway, the yield PCA from FA can directly undergo an enzymatic aromatic ring-opening reaction, but benzoate from CA cannot. The benzoate, which resulted from CA, should be converted into a derivative of catechol or other diphenol compounds before it can be ring-opened for further degradation. Strains M1N1S27, M4R2S49^T^, HR5S1, and M1R5S2^T^ harbored a *benABCD* operon, which was responsible for the direct conversion of benzoate to catechol. The transcriptomic analyses showed that *benABCD* was significantly upregulated by CA and downregulated by FA in strains M4R2S49^T^ and M1R5S2^T^. In addition, a phenylacetyl-CoA epoxidase gene operon (*paaABCDE*), which was responsible for the reduction of phenylacetyl-CoA (PA-CoA) to form 1,2-epoxyphenylacetyl-CoA, was found in all five strains. However, this operon was upregulated by CA in M4R5S9^T^ and downregulated by CA or FA in strains M4R2S49^T^ and M1R5S2^T^. This may be an alternative pathway for the conversion of benzoate to catechol in strain M4R5S9^T^. In addition, there are many other hydroxylase genes in these five strains. A *p*-hydroxybenzoate 3-hydroxylase gene (*pobA* or *praI*) located in the *pca* operon was found in all five strains, indicating that all five strains could convert PHA to PCA. Furthermore, strains M4R2S49^T^, M1N1S27, M4R5S9^T^, and M1R5S2^T^ harbored the *m*-hydroxybenzoate 4-hydroxylase gene (*mobA*), which enables the host to degrade *m*-hydroxybenzoate to PCA. In addition, strains M4R5S9^T^, HR5S1, and M1R5S2^T^ could convert *m*-hydroxybenzoate to gentisic acid catalyzed by 3-hydroxybenzoate 6-hydroxylase (XlnD) (Figure 3). However, all these hydroxybenzoate hydroxylase genes were not upregulated by FA or CA, that because these genes were not directly involved in the degradation of FA and CA.

Aromatic ring fission is another key step during the degradation of aromatic compounds. The genome analyses revealed that all five strains harbored several gene clusters involved in the ring-cleavage of aromatic compounds. A *pca* gene cluster, which includes *pcaRJIFCDBGH* and is involved in the degradation of PCA, was annotated in all five strains. Specifically, the ring of PCA, which was obtained from FA or the hydroxylation of PBA, was then opened due to the catalysis of protocatechuate 3,4-dioxygenase (PcaGH). The produced straight chain dicarboxylic acid was subsequently degraded to succinyl-CoA and acetyl-CoA after consecutive reactions, which were catalyzed by the enzymes encoded by the *pca* cluster (Figure 3b). Although all five strains harbored the *pca* gene cluster, there were some minor differences among them. Strains M4R5S9^T^, M1N1S27, and M1R5S2^T^ completely converted 4-carboxymuconolactone to 3-oxoadipate using two independent enzymes (PcaC and PcaD), while that in strains M4R2S49^T^ and HR5S1 was completed by a fusion enzyme PcaC (Figure 3a). In addition, all five strains still harbored other ring-fission genes, such as that for catechol 1,2-dioxygenase (C12O, including *catA* and *benA*), and the gene for catechol 2,3-dioxygenase (C23O). It is notable that two catechol 1,2-dioxygenae genes (namely, *catA* and *catA2*) and a catechol 2,3-dioxygenase gene were significantly upregulated by CA, and downregulated by FA in the three strains. In contrast, *pcaGH* was upregulated by FA, but downregulated by CA. This suggests that these strains employ two different pathways to degrade CA and FA after the removal of the acryl group from the aromatic ring via CoA-dependent non-β-oxidation. *pcaGH* was responsible for the ring-opening of the PCA produced from FA, while *catA* or *C23O* genes, which were not located in the *pca*-operon, were responsible for the ring fission of catechol that resulted from CA.

In addition to the ring-fission pathway that utilized catechol or its derivatives as an intermediate, the usage of gentisic acid as the substrate was another ring-fission pathway. Three of the five novel isolates harbored gentisate 1,2-dioxygenase (GdoA), by which the host could convert gentisic acid to 3-maleylpyruvate. The aromatic ring-opened product 3-maleylpyruvate was subsequently degraded to pyruvate and fumarate, which can be thoroughly degraded or transformed into other chemicals as a carbon skeleton for growth through the TCA cycle (Figure 3b). However, not all these genes were significantly upregulated by both PAs, indicating that the pathways represented by these genes were not involved in the degradation of either compound.

**Figure 4:**
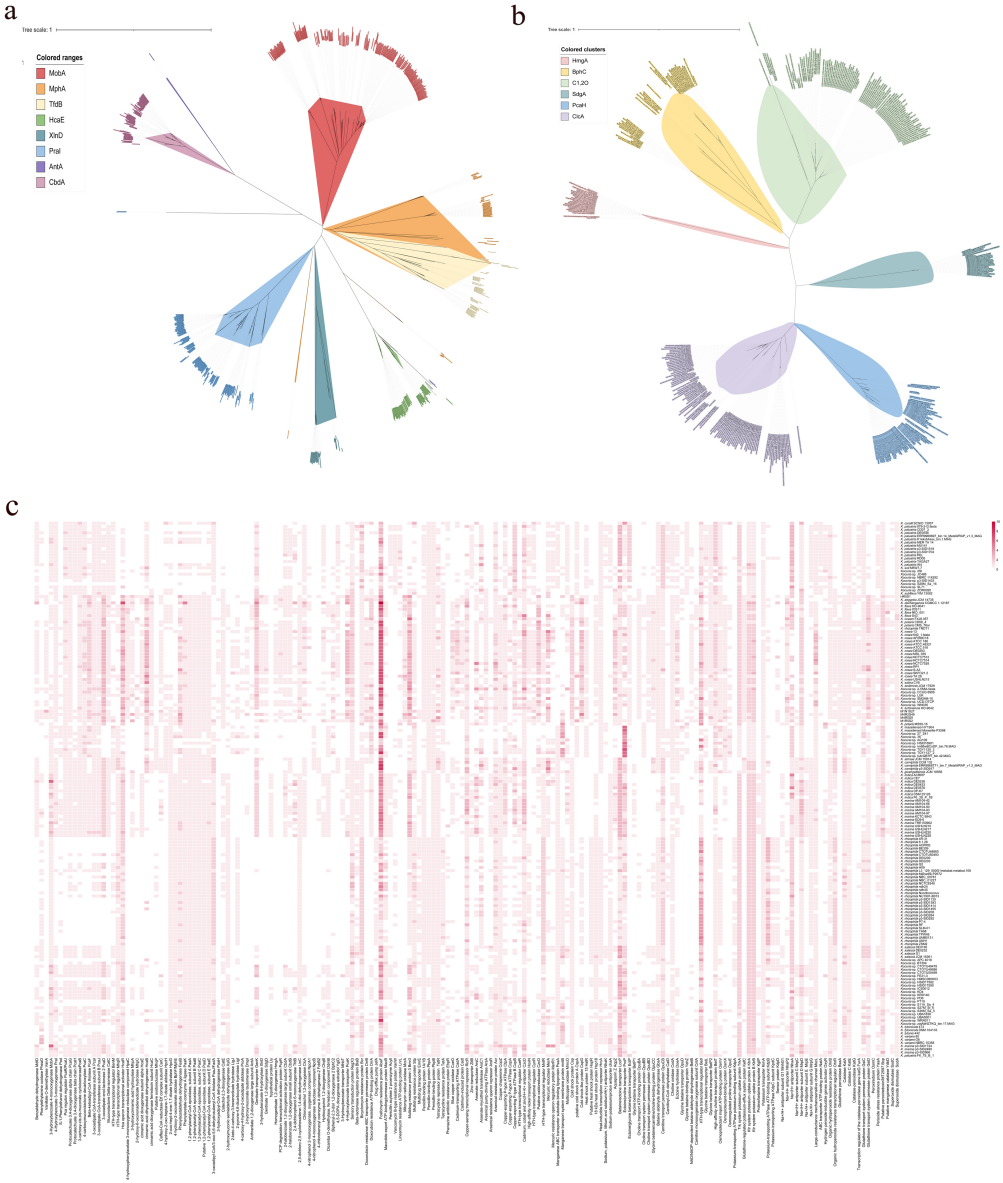
The phylogenetic trees based on aromatic compounds hydroxylase **(a)** and aromatic ring-opening dioxygenase **(b)**, and the distribution of enzymes relating to aromatic compounds degradation, antibiotic resistance, osmotic balance maintenance, and oxidative stress defense **(c)**.

In addition to the CoA-dependent non-β-oxidation, CA can also be directly dioxygenated at the 2- and 3-sites of the aromatic ring, which was consecutively catalyzed by HcaA1A2CD and HcaB. The production of 2,3-dihydroxy-CA was then ring-opened by MphB. In the three strains whose transcriptomes were analyzed, the *hcaB* genes were upregulated by CA and downregulated by FA, suggesting that the CA-2,3-dioxygenation may also be a branch degradation pathway of CA. However, no *hcaA1A2CD* genes or *mphB* gene were found in all these genomes (Figures 3, 4).

As the transportation of the PAs, a 4-hydroxybenzoate transporter gene (named *pcaK*) was consistently upregulated by FA but downregulated (in strains M4R5S9^T^ and M4R2S49^T^) or upregulated at a low level (in strains M1R5S2^T^) by CA. This suggests that this gene may play a key role in the uptake of FA by these strains. In contrast, two genes, *genK* encoding the gentisate transporter and *benE* (benzoate/H + symporter), were upregulated by CA but downregulated by FA in strains M4R2S49^T^ and M1R5S2^T^, suggesting that they may play vital roles during the transmembrane absorption of CA. In strain M1R5S2^T^, three transporter genes (namely, *sotB*, responsible for sugar efflux transporter, and two inner membrane transporter genes: *ydhP* and *yhjE*) were all significantly upregulated by both PAs.

### 3.4 Comparative genome feature of the genus *Kocuria*

A comparative genomic analysis showed that the 168 selected *Kocuria* genomes contained 42,983 gene families, and the numbers of core, accessory, and unique genes were 231, 29,888, and 12,864, respectively. According to Heaps’ law, the pan-genome of *Kocuria* remains open (*b* = 0.44), indicating that with each newly added genome, the number of new genes increases the genetic repertoire of the species (Supplementary Figure S1).

All strains of *Kocuria* harbored the glycolysis/gluconeogenesis pathway, the pentose phosphate pathway, and the citrate cycle pathways, indicating that all strains of *Kocuria* could utilize glucose, fructose, and even starch as their carbon source for growth. In terms of the biosynthesis of amino acids, the genus strains can synthesize all types of amino acids except tryptophan with the simple substrates from carbohydrate metabolism. Majority of the *Kocuria* strains harbored various genes involved in the reduction of nitrate, nitrite, and sulfate (Supplementary Figure S2). These metabolic abilities facilitate the case in which the *Kocuria* strains can obtain carbon, nitrogen, sulfur, and energy from the environment, thereby enhancing their adaptability to diverse environments.

It was also revealed that all the strains of *Kocuria* harbored the betaine aldehyde dehydrogenase gene *gbsA*, carnitinyl-CoA dehydratase *caiD*, ectoine hydrolase *doeA*, and choline oxidase gene *coda* (Figure 4c). However, only seven *ectB* genes and two *ectC* genes, which are two key genes involved in the biosynthesis of ectoine, were found in these strains. No *ectA* gene was found, indicating that the *Kocuria* strains cannot synthesize ectoine as an osmoprotectant by themselves under hypersaline conditions. However, many osmoprotectant-binding proteins and transporter genes were found in these 168 genomes. For example, a total of 319 betaine/ectoine transporter gene *lcoP*, and 265 betaine/proline transporter gene *proP* were annotated in 155 and 140 *Kocuria* strains, respectively. In addition, majority of them could bind and transport the choline via a high-affinity choline transporter BetT (223 genes). Some of the *Kocuria* strains could absorb proline through the osmoprotectant proline transporter OsmX. The accumulation of potassium in the cytoplasm is another crucial mechanism for maintaining osmotic balance. Many *ktrAB* (296 and 303 genes from all 168 strains, respectively), *trkA* (309 genes from 163 strains), and *kimA* (164 genes from 164 strains), were annotated in the genomes of these strains. Furthermore, the *Kocuria* strains also harbored Na^+^/H^+^ antiporter genes, by which the cells could export sodium. These results indicate that the *Kocuria* strains could maintain the osmotic balance via several mechanisms.

Many PAs-degrading genes are ubiquitous in the *Kocuria* genomes. For example, a total of 185 *mobA* genes were annotated in 142 *Kocuria* genomes, indicating that majority of the *Kocuria* strains possess the ability to degrade *m*-hydroxybenzoate. A total of 116 genomes contained the *praI* (or named *pobA*) gene. Furthermore, 136 *Kocuria* strains harbored at least one gene of the *pca* cluster, indicating that the biodegradation of PCA via the β-ketoadipic acid pathway was quietly present in the genus *Kocuria*. In addition to the β-ketoadipic acid degradation pathways, 91 protocatechuate-4,5-dioxygenase genes (or catechol 2,3-dioxygenase genes) were found in 90 *Kocuria* strains. A total of 31 *xlnD* and 27 *sdgD* genes were identified in 28 and 26 *Kocuria* strains, respectively, indicating that these strains could degrade *m*-hydroxybenzoate via the gentisate-degrading pathway. Majority of the *Kocuria* strains harbored *paa* clusters, indicating that these strains could degrade phenylacetate via the *Paa*-degradation pathways. It is notable that 59 genomes of *Kocuria* harbored *hcaE* genes, a key enzyme that catalyzes the dioxygenation reaction of cinnamic acid to 2,3-diohydro-2,3-dihydroxycinnamic acid. A total of 167 *hcaB* genes, which were involved in the reaction of 2,3-dihydro-2,3-dihydroxycinnamic acid to 2,3-dihydroxycinnamic acid, were found in 68 strains of *Kocuria*. No *mohB* gene, which catalyzes the aromatic ring-cleavage reaction, was annotated in the genomes (Figure 4). However, several potential enzymes could substitute for MohB. For example, *bphC*, a gene involved in the cleavage of aromatic rings, was found in Majority of the *Kocuria* strains. The ring-opened product could be subsequently degraded to the small molecule dicarboxylic acid, which was catalyzed by MhpC or the other isoenzymes (Figure 4c). The results of comparative genomic analysis revealed that strains of the genus *Kocuria* generally have multiple pathways to degrade PAs and other aromatic compounds, indicating the great application potential of the genus *Kocuria* in bioremediation.

## 4 Discussion

In this study, five haloalkaliphilic *Kocuria* strains were isolated from the rhizosphere and bulk saline soils of the halophyte *K. cuspidatum*. All five strains were able to degrade several PAs at pH 8.0 in the presence of a high concentration of salt. It is generally believed that the *Kocuria* strains have a strong ability to degrade pollutants and tolerate abiotic stress. Not only they degrade organic pollutants, such as fuel oil or heavy oil (Huang et al., 2012; Promsing et al., 2021), and Aflatoxin B1 (Wang and Nan, 2025), they can also tolerate heavy metals and high concentration of salt. However, only *Kocuria* sp. TIBETAN4, a strain sourced from a saline lake located in northwestern China, could degrade phenol via the ortho-pathway under saline-alkaline conditions (Wu et al., 2018). There is less information on the degradation of PAs by the *Kocuria* strains. To the best of our knowledge, this is the first report about the degradation of PAs by *Kocuria* strains at saline-alkaline conditions. This should facilitate their use as agents for the bioremediation of saline-alkaline environments contaminated with PAs.

Although all five strains could degrade CA, FA, and some other PAs, they exhibited significant differences in the degradation rates of CA and FA. These differences may be attributed to the diversity of PA-degrading pathways. The genomic annotation and transcriptomic analyses revealed that all five strains utilized the CoA-dependent non-β-oxidation, protocatechuate 3,4-dioxygenation ring-open, and the β-ketoadipic acid pathway to degrade FA. After the conversion of CA to benzoate, some of them utilized the BenABCD enzyme system to convert benzoate to catechol; however, strain M4R5S9^T^ did not harbor the *ben*-operon. In strain M4R5S9^T^, the *paa*-operon genes were upregulated by CA and downregulated by FA. No other pathway genes were significantly upregulated by CA. In strains M4R2S49^T^ and M1R5S2^T^, the genes in the *paa* operon were not upregulated by both PAs. These findings suggest that strain M4R5S9^T^ may select the *paa* pathway as its alternative way to degrade CA, owning to its inability to convert benzoate to catechol. These may explain why CA was slowly degraded by strain M4R5S9^T^. Notably, all five strains may select PcaGH dioxygenases for the aromatic-ring cleavage of PCA from the degradation of FA and non-PcaGH dioxygenases (CatA and C23O) for the ring-cleavage of benzoate from the degradation of CA. These findings indicated that CatA was primarily responsible for the ring-opening of catechol or its derivatives, which lack a carboxyl group on the aromatic ring. At the same time, the PcaGH was primarily responsible for the degradation of PCA or its derivatives. Naturally, the genes in the *pca* operon were also responsible for the degradation of the ring-opening products of PCA. While the β-ketoadipic acid pathway, which was not encoded by a gene within the *pca* operon was utilized for the degradation of catechol originating from CA. The presence of these multiple enzyme systems may improve the efficiency of bacterial strain metabolism of PAs in various environments.

**Figure 5:**
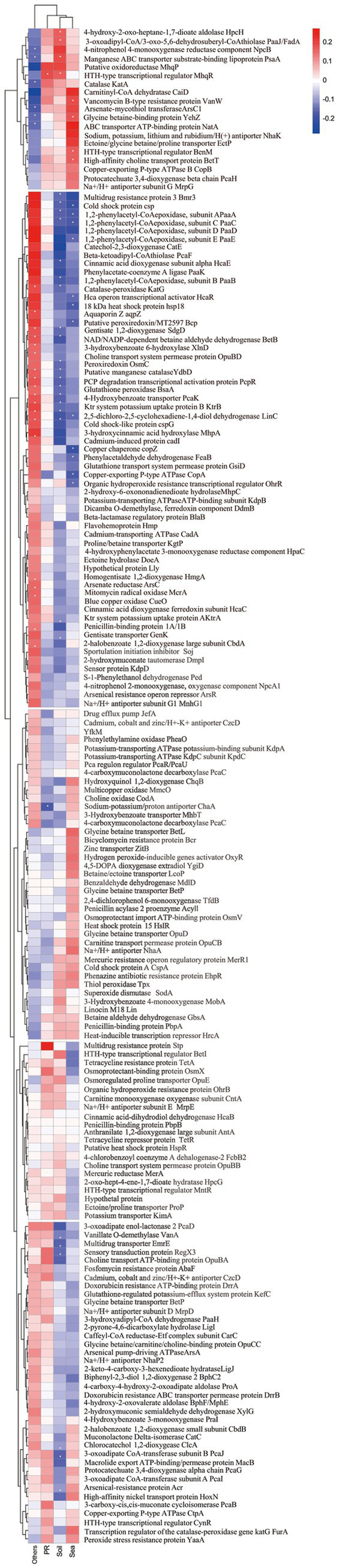
Correlation of the gene distribution and the strain isolation source.

The comparative genomic analysis revealed that the majority of *Kocuria* strains universally harbored PAs-degrading genes, such as the aromatic ring-opening genes, *pcaGH*, *sdgD*, the C23O gene, and *bphC*. As the monomers of lignin, the PAs are abundant in soils inhabited by these bacteria (Xie et al., 2023). The powerful PA-degrading abilities endow the *Kocuria* strains with higher competitiveness, which widens the territory in which they can survive. Alternatively, the PAs are also the most common allelochemicals in the soils, particularly in the rhizosphere (Peracchi et al., 2024). Low concentration of PAs could promote the growth and development of the host plant, while high concentrations may inhibit their growth and decrease the rate of seed germination (Li et al., 2015). Therefore, the *Kocuria* strains in the rhizosphere may play an important role in regulating the concentration of PAs in the rhizosphere environment, resulting in beneficial effects on the host plant. The correlation analysis between the genes and environments revealed that many of the genes were highly related to the isolation source of the host. Many of the aromatic compounds-degrading genes were positively related to the rhizosphere habitats, including phenylacetaldehyde dehydrogenase gene *feaB*, 3-dydroxyadipyl-CoA dehydrogenase *paaH*, 2-hydroxymuconate tautomerase *dmpI*, 3-hydroxybenzoate transporter *mhbT*, dicamba o-demethylase *ddmA*, *bphC*, 4-hydroxyacetophenone monooxygenase *hapE*, and several genes related to the degradation of phenylacetate (Figure 5). In addition, *hipO*, *npcA*, and *vanA*, 4-hydroxybenzoate transporter gene *pcaK*, 3-hydroxycinamic acid hydroxylase *mhpA*, and hydroxybenzoate 6-hydroxylase gene *xlnD* were positively related to the plant rhizosphere and soil, and negatively associated with seawater and the other sources, indicating the higher abundance in terrestrial ecological environments (Figure 5). These findings imply that the existence of these genes may be the result of coevolution between bacterial strains and specific environments.

The calculation for the analysis of the habitats, from which the strains were isolated, showed that many of *Kocuria* strains originated from the hypersaline habitats, such as seawater, and saline-alkaline soil (Figure 1a). However, less research has been conducted on the mechanism of tolerance to salt stress tolerance in this genus. The five *Kocuria* strains were able to degrade the PAs under a moderate saline condition, which once again confirms the extremely high environmental adaptability of the genus. Bacteria usually adopt two strategies to respond to osmotic stress. The first is to accumulate organic osmoprotectants and potassium, while the second is to export the sodium (Sunita et al., 2020; Tsujii et al., 2020). Glycine-betaine and ectoine are the two most common organic osmoprotectants in halophilic bacteria. This study revealed that *Kocuria* strains primarily depend on the absorption of osmoprotectants from their environments to maintain osmotic balance, rather than producing them through biosynthesis. However, majority of the genus strains harbored multiple potassium ion absorption channel genes, including *ktrAB*, *trkA*, and *kimA*. In addition, the strains harbored Na^+^/H^+^ antiporter genes, which enabled the cells to export sodium. During the degradation test, the medium (MMSM) does not contain organic osmoprotectants, but it contains sufficient potassium ions. These results suggest that absorbing potassium ions and expelling sodium ions is a major way for the *Kocuria* strains to maintain their osmotic balance. The comparative genomic analysis also revealed that majority of the *Kocuria* strains harbored the bicyclomycin resistance gene *bcr* (232 genes distributed across 163 genomes) and a total of 671 of the fosfomycin resistance gene *abaF*. The penicillin-binding protein genes *ponA* (146 genes in 146 genomes), *pbpA* (136 genes in 136 genomes), and *pbpB* (168 genes in 166 genomes), and the tetracycline resistance gene *tetA* (110 genes in 116 genomes) and its regulator gene *tetR* (170 genes in 144 genomes) were also found in the majority of *Kocuria* strains (Figure 4c). A total of 281 multidrug resistance protein 3 genes (*bmr3*) were found in 132 *Kocuria* strains. These genes endow the genus with the universal ability to resist antibiotics, facilitating their colonization in the rhizosphere. In terms of heavy metal resistance, many genes that encoded resistance to heavy metals were also annotated in the *Kocuria* strains. For example, 214 *acr* genes (in 161 genomes), which were responsible for arsenical resistance, were annotated in these strains. In addition, 118 *arsC* genes encoding arsenate reductase were found in 94 genomes, indicating that *Kocuria* strains universally harbor arsenate resistance. A total of 150 mercuric reductase genes, *merA*, were annotated in these 90 *Kocuria* genomes (Figure 4c). In addition, oxidative stress within the cells is the direct reason why cells die in various extreme environments. All of the *Kocuria* strains harbored at least one way to remove the oxidative molecules in time, including organic hydroperoxide resistance gene (*ohrB*, 151 genes in 148 strains) and its regulator gene (*ohrR*, 172 genes in 134 strains), *katA* (187 genes in 164 strains) and *katE* (160 genes in 159 strains), and *sodA* (169 genes from 166 strains). Furthermore, the *Kocuria* strains also harbored other genes for removing oxidative agents. In all these manners, the *Kocuria* strains could adapt better to various stresses in the environment, undoubtedly increasing the abundance of bacterial strains in extreme habitats and the remediation efficiency of the PA-contaminating environment, and the efficiency of colonization in the rhizosphere of plants. These findings of comparative genomic analysis can provide important guidance for the stimulation of the genus *Kocuria* in the environment.

**Figure 6:**
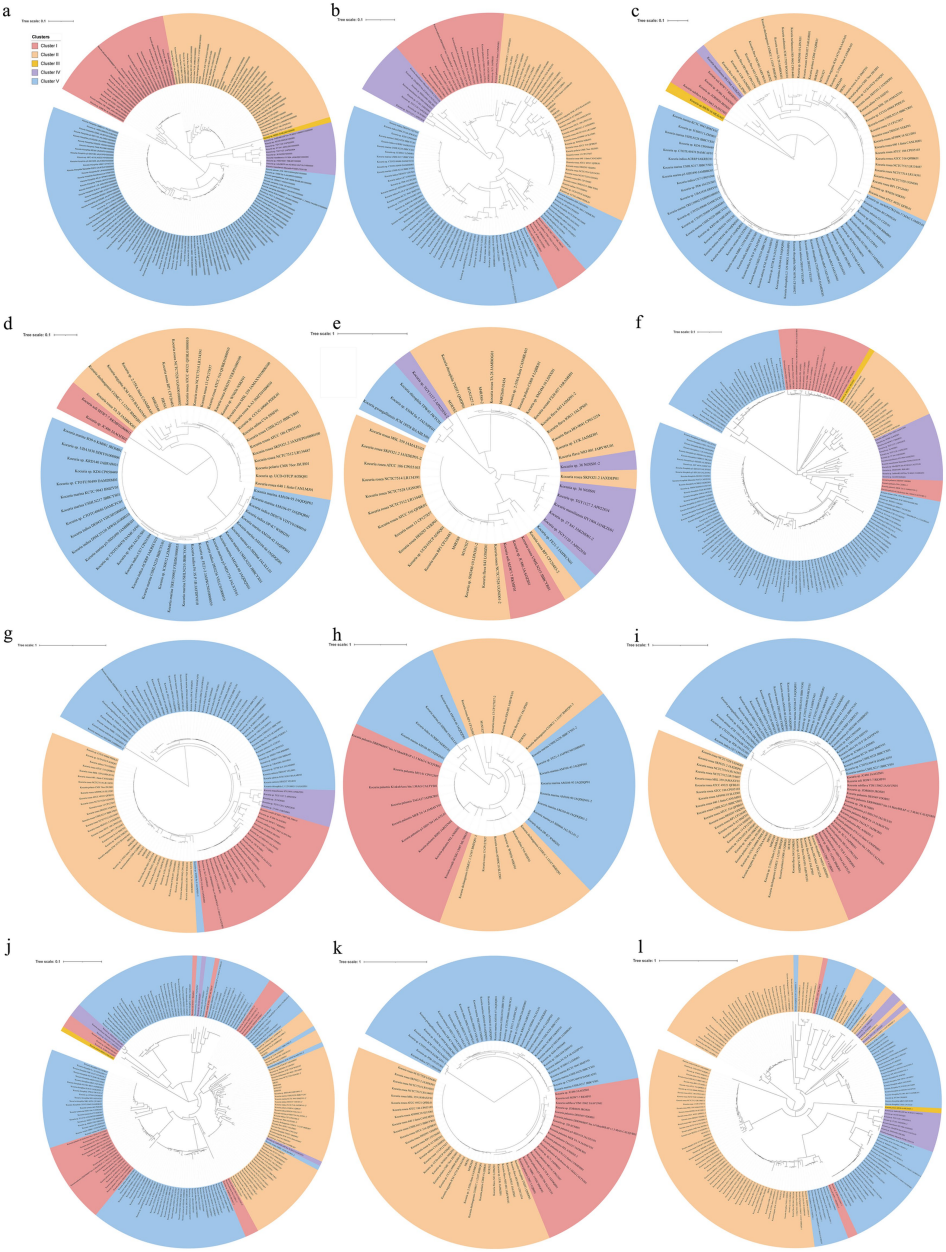
Phylogenomic tree of *Kocuria* strains (including non-type strains) based on core genome using GTDB **(a)**, and the phylogenetic trees based on the single amino sequence of PcaG **(b)**, CatE **(c)**, HcaE **(d)**, BphC **(e)**, MobA **(f)**, PraI **(g)**, TfdB **(h)**, CbdA **(i)**, Acr **(j)**, CzcD **(k)**, and HcaB **(l)**. The five clusters were subdivided according to the topological relation on the phylogenomic tree.

The analysis described above naturally leads to a question: how and when did these *Kocuria* strains acquire their high PA-degrading abilities? Bacteria primarily acquire genetic resources through two pathways. The first pathway is to acquire them from their ancestry cells, which is known as vertical gene transfer (VGT), while another way is to obtain the genes from the other cells or other organisms by transformation or transfection, which is called horizontal gene transfer (HGT) (Coleman et al., 2021). The phylogenomic analysis showed that these strains of *Kocuria* could be subdivided into five clusters. Cluster I includes *Kocuria subflava*, *Kocuria coralli*, *Kocuria soli*, *Kocuria palustris*, and some unidentified species of *Kocuria*. Cluster II is composed of *Kocuria flava*, *Kocuria dechangensis*, *Kocuria sediminis*, *Kocuria turfanensis*, *Kocuria oceani*, *Kocuria segyptia*, and *Kocuria salina*, the three novel species identified in this study, and the type species *K. rosea*, as well as a misidentified *Kocuria rhizophilia* strain. Cluster III only contains the misidentified strain *K. rosea* subsp. *polaris* MS50-16. Cluster IV consists of “*K. massiliensis*” and eight unidentified *Kocuria* strains, while Cluster V primarily consists of *Kocuria atrinae*, *Kocuria gwangalliensis*, *Kocuria carniphila*, *Kocuria marina*, *Kocuria indica*, *Kocuria Marina*, *Kocuria varians*, *Kocuria salsicia*, *Kocuria Tytonicola*, and some unidentified *Kocuria* strains (Figure 6). The topology of the phylogenetic tree based on a single amino acid sequence of PcaG, CatE, BphC, PraI, TfdB, CbdA, and MobA was primarily similar to the phylogenomic tree, suggesting that these genes were mostly obtained from VGT. However, those based on the amino acid sequence of Acr and HcaB were quite different from that of the phylogenomic tree, suggesting these genes may have been obtained via HGT. In addition, some genes, such as *hgmA* and *sdgA*, were only present in the Cluster II strains (Figure 4), indicating that the *Kocuria* strains in Cluster II possess additional pathways to degrade aromatic compounds compared to the other four clusters. It is also revealed that the members of Cluster II have a high proportion that harbor genes to degrade aromatic compounds (Figure 6). This may be because the common ancestor of these clusters accidentally obtained the genes from other organisms by HGT.

## 5 Conclusion

A total of five halotolerant and halophilic *Kocuria* strains were isolated from the rhizosphere soil and bulk saline soil of the euhalophyte *K. cuspidatum*. The five strains were identified as three novel species of *Kocuria* using a polyphasic taxonomy analysis, namely, *K. rhizosphaerae* sp. nov. (type strain M1R5S2^T^), *K. kalidii* sp. nov. (type strain M4R5S9^T^; non-type strain M1N1S27), and *K. rhizosphaericola* sp. nov. (type strain M4R2S49^T^; non-type strain HR5S1). All five strains could degrade FA and CA as their sole carbon and energy source for their growth under saline-alkaline conditions. The genomic and transcriptomic analyses revealed that all five strains degraded FA via the CoA-dependent non-β-oxidation pathway, and protocatechuate 3,4-dioxygenase and the β-ketoadipic acid pathway, while they degraded CA via the CoA-dependent non-β-oxidation pathway, benzoate 1,2-dioxygenase for conversion of benzoate to catechol, catechol 1,2-dioxygenase or catechol 2,3-dioxygenase for ring fission, and the β-ketoadipic acid pathway. A comparative genomic analysis revealed that PAs-degrading genes, including PAs hydroxylase or dioxygenase genes, and catechol and its derivative ring-opening genes are universal in the *Kocuria* strains. In addition, the *Kocuria* strains could maintain their osmotic balance by absorbing organic osmoprotectants from the environment, taking up potassium through multiple pathways, and effluxing sodium through Na^+^/H^+^ antiporter channel. This study illustrates the mechanisms that are utilized by the *Kocuria* strains to adapt to extreme environmental condition and rhizosphere of halophyte, and provide important guidance for the stimulation of the genus of *Kocuria* in the environment, and demonstrates the enormous potential for the application of these strains in the remediation of PA pollution in saline-alkaline environments and their pivotal roles of *Kocuria* strains on PA degradation in ecosystem.

## Data Availability

The datasets presented in this study can be found in online repositories. The names of the repository/repositories and accession number(s) can be found in the article/Supplementary material.

## Footnotes

^1^ https://blast.ncbi.nlm.nih.gov
