# Supplementary material for: Multiple-omics analysis of three novel haloalkaliphilic species of Kocuria revealed that the phenolic acid-degrading abilities are ubiquitous in the genus
Source: Front Microbiol. 2025 Jul 30;16:1626161. doi: 10.3389/fmicb.2025.1626161 (PMC12343579; doi:10.3389/fmicb.2025.1626161)

Supplemental materials for manuscript

Multiple-omics analysis of three novel haloalkaliphilic species of *Kocuria* revealed the phenolic acids-degrading abilities are ubiquitous in the genus

Lian Xu[1,2], Rui-Qi Sun[2], Jia-Hui Zeng[2], Hua-Mei Wei[2], Biao Shen[1], Ji-Quan Sun[2*]

1 Jiangsu Key Lab for Organic Solid Waste Utilization, Educational Ministry Engineering Center of Resource-saving Fertilizers, Jiangsu Collaborative Innovation Center for Solid Organic Waste Resource Utilization, Nanjing Agricultural University, Nanjing, 210095, P. R. China

2 Ministry of Education Key Laboratory of Ecology and Resource Use of the Mongolian Plateau, School of Ecology and Environment, Inner Mongolia University, Hohhot, 010021, P. R. China

*Correspondence author

Ji-Quan Sun, Department of Ecology, School of Ecology and Environment, Inner Mongolia University, Hohhot 010021, PR China, Tel/Fax: +86-471-4991436. E-mail address: sunjq81@163.com

Table S1 Characteristics of the genomes sequence used in present study.

Strains	Accession No.	G+C content (%)	Genome size	Ns	Scaffolds	N50 (scaffolds)	Coding density (%)	Predicted genes	Complet-eness	Contami-nation
HR5S1	JBISWN01	72.1	4.01	0	110	98939	87.8	3741	99.3	0
K. aegyptia JCM 14735	BAAAOA01	72.4	3.86	851	63	187666	87.5	3578	99.9	0.88
K. atrinae JCM 15914	BAAAQA01	63.9	3.15	759	52	168433	90.0	2830	99.3	0
K. carniphila CCM 132	NOIT01	64.2	3.42	0	49	158971	89.7	3055	100.0	0
K. carniphila ERR9968771_bin.7_MetaWRAP_v1.3 _MAG	CAUREL01	64.1	3.26	0	56	101669	90.0	2912	99.3	0
K. carniphila p3-SID917	JALXKX01	64.1	3.37	0	161	50327	89.9	3040	100.0	0
K. coralli SCSIO 13007	ML708610	68.6	3.43	42	71	188027	89.9	3022	100.0	0
K. dechangensis CGMCC 1.12187	BMEQ01	72.3	4.46	290	91	139803	87.3	4152	99.3	0
K. flava HO-9041	CP013254	73.9	3.64	0	5	3504335	88.3	3242	99.3	0.66
K. flava IOS11	JALJPS01	74.3	3.47	614	88	63998	89.0	3100	99.3	2.65
K. flava NIO_001	JARVWU01	74.4	3.43	0	9	508879	89.0	3043	100.0	0
K. flava S43	LOMZ01	73.4	3.90	0	6	3548480	86.6	3558	98.3	0.66
K. gwangalliensis JCM 18958	BAABLN01	64.9	3.16	19	71	174659	89.5	2880	100.0	0
K. indica ACRRP	JAKREC01	68.7	2.91	0	80	99155	88.1	2686	99.3	0
K. indica CE7	CP035504	68.7	2.84	0	2	2809846	88.2	2565	98.0	0
K. indica DE0236	VELC01	68.8	2.90	192	58	354133	88.3	2668	99.3	0
K. indica DE0433	VDUS01	68.7	3.09	193	56	358894	88.3	2808	99.3	0.66
K. indica DE0576	VDTY01	68.8	2.88	108	89	161550	88.6	2596	99.3	0.66
K. indica DP-K7	WMHZ01	69.0	3.01	0	57	156175	89.5	2709	98.7	0
K. indica DSM 25126	MWSL01	68.9	2.88	0	48	135131	88.9	2614	99.3	0.66
K. indica F6_3S_P_1B	JAFDPV01	68.8	2.80	106	27	245667	88.7	2524	99.3	0.66
K. marina AM104-42	JAQDPS01	68.9	3.07	140	77	115183	89.1	2735	98.7	0
K. marina AM104-66	JAQDQD01	69.1	3.01	60	53	178694	89.0	2684	98.7	0.66

Strains	Accession No.	G+C content (%)	Genome size	Ns	Scaffolds	N50 (scaffolds)	Coding density (%)	Predicted genes	Complet-eness	Contami-nation
K. marina AM104-90	JAQDQN01	68.9	3.12	40	57	229660	89.1	2751	98.7	0.66
K. marina AM104-93	JAQDQP01	68.9	3.13	70	60	210306	89.0	2735	98.7	0.99
K. marina AM104-97	JAQDQR01	68.7	3.17	30	77	180114	88.9	2808	98.7	0
K. marina KCTC 9943	BMZV01	68.8	2.79	47	21	580827	88.7	2513	98.7	0.66
K. marina p3-SID1724	JALXTF01	69.0	2.88	0	130	42102	89.0	2639	98.0	0
K. marina p3-SID2490	JAMBBG01	68.8	2.80	0	97	51950	88.8	2549	98.7	0
K. marina p3-SID866	JALXLL01	69.2	2.90	0	116	46077	89.5	2606	98.7	0
K. marina SO9-6	JROM01	68.8	3.07	0	62	183121	88.5	2777	99.3	0
K. marina TRE150902	FXBD01	68.9	2.86	0	54	98614	88.0	2714	96.9	0
K. marina USHLN210	JBBCYO01	68.6	2.99	0	54	217351	88.5	2751	98.7	0
K. marina USHLN217	JBBCYS01	68.8	2.88	0	39	329263	88.4	2623	99.3	0
K. marina USHLN220	JBBCYU01	68.8	2.89	0	36	371620	88.4	2618	98.7	0
K. marina USHLN228	JBBCYX01	68.8	2.85	0	31	384214	88.7	2562	98.7	0
K. massiliensis HY1904	JANKZE01	64.9	2.73	0	16	405026	90.0	2411	98.8	0
K. massiliensis Marseille-P3598	LT835158	61.0	2.43	160	4	1484501	89.9	2151	99.1	0
K. oceani FXJ8.057	JARAMH01	72.4	4.24	0	63	241667	87.7	3961	99.3	0
K. palustris 879-2-G	CANMNH01	70.3	2.93	0	80	182322	88.5	2601	99.3	0
K. palustris CD07_3	LQBJ01	70.4	2.83	19	18	416347	88.8	2467	99.3	0.33
K. palustris DE0549	VDOR01	72.1	2.82	33	61	172008	89.0	2422	99.3	0
K. palustris ERR9968897_bin.14_ MetaWRAP_v1.3_MAG	CAUQVR01	70.5	2.75	0	166	25205	89.2	2511	97.0	0.99
K. palustris F6_7S_B_1	JAFDQD01	70.5	2.84	20	34	191187	88.7	2510	99.3	0
K. palustris K1ekxbAssx_bin.1.MAG	CALTYS01	70.5	2.88	0	78	55105	89.3	2547	99.0	0.33
K. palustris MER TA 14	JAMAVY010	70.5	2.86	0	70	307465	88.7	2549	99.3	0.33
K. palustris MU141	CP012507	70.5	2.85	0	1	2854447	88.7	2482	99.3	0

Strains	Accession No.	G+C content (%)	Genome size	Ns	Scaffolds	N50 (scaffolds)	Coding density (%)	Predicted genes	Complet-eness	Contami-nation
K. palustris p3-SID1518	JALXUU01	70.4	2.81	0	146	30790	88.9	2481	98.7	0
K. palustris p3-SID1704	JALXTL01	70.3	2.89	0	217	19818	89.4	2552	97.6	0.33
K. palustris PEL	ANHZ02	70.4	2.87	0	55	140153	88.9	2535	99.3	0
K. palustris RD05	JAROYN01	70.1	2.96	0	141	36101	88.2	2709	99.3	0.99
K. palustris TAGA27	JAFBCR01	70.2	2.90	0	1	2897960	88.3	2510	99.3	0
K. palustris W4	CZJR01	69.9	3.09	0	95	125991	88.0	2779	99.3	1.21
K. rosea subsp. *polaris* CD08_4	LQBK01	72.6	3.83	32	42	221530	88.9	3443	98.7	0
K. rosea subsp.*polaris* CMS 76or	JSUH01	72.8	3.78	2	67	238548	89.4	3458	99.2	0
Kocuria sp. MS50-16	MLDA01	68.3	3.43	0	51	127707	91.0	3163	99.8	1.76
K. rhizophila 4R-31	SPNK01	70.7	2.66	0	26	245819	88.9	2297	98.7	0
K. rhizophila 6.1.26	JANLGN01	70.7	2.69	158	85	51248	88.7	2343	98.7	0
K. rhizophila ACRRQ	JAKRED010	70.6	2.82	0	49	97549	89.1	2479	98.4	0
K. rhizophila BE309	JAVDXP01	70.7	2.66	0	19	282561	89.0	2282	98.7	0
K. rhizophila CTOTU48865	DAMBGN01	70.6	2.83	200	16	324475	88.9	2478	98.7	0.05
K. rhizophila CTOTU50493	DAMDIG01	71.4	2.65	630	93	45447	88.6	2390	98.0	0.66
K. rhizophila DE0200	VEDQ01	70.4	2.93	17	169	526285	88.7	2648	98.7	0
K. rhizophila DE0203	VEDN01	70.6	2.75	107	160	365936	88.1	2421	98.0	0
K. rhizophila G2	CZJW01	70.8	2.88	0	87	88861	87.8	2558	98.7	0.99
K. rhizophila H09	JAEOAT01	71.2	2.72	0	117	38104	88.3	2406	98.0	0
K. rhizophila L3_129_000G1metabat.metabat.108	JAHAHP01	70.3	2.79	0	84	55434	89.1	2541	97.7	1.43
K. rhizophila Marseille-P2672	CAJFZU01	70.7	2.69	1	43	135790	88.5	2310	98.7	0
K. rhizophila NBC_00781	CP108927	71.0	2.77	0	1	2773374	87.8	2411	98.7	0
K. rhizophila NBC_01227	CP108528	70.6	2.74	0	1	2735685	88.4	2366	98.7	0
K. rhizophila NCTC8340	LR134409	71.2	2.70	0	1	2697831	88.1	2316	98.7	0

Strains	Accession No.	G+C content (%)	Genome size	Ns	Scaffolds	N50 (scaffolds)	Coding density (%)	Predicted genes	Complet-eness	Contami-nation
K. rhizophila ndh24	JAEUXN01	71.3	2.69	52	45	150949	88.4	2354	99.3	0
K. rhizophila ndh45	JAEUXO01	71.3	2.74	49	59	103878	88.4	2376	98.5	0
K. rhizophila Noodlococcus	CP097204	70.6	2.73	0	1	2732108	88.1	2357	98.7	0
K. rhizophila NU1901-B013	JADMKX01	71.2	2.68	500	2	2668985	87.5	2314	98.7	0
K. rhizophila p3-SID1135	JALXXE01	70.8	2.63	0	72	54042	89.2	2285	96.7	0
K. rhizophila p3-SID1383	JALXVP01	70.8	2.66	0	79	48584	89.0	2284	97.7	0
K. rhizophila p3-SID1414	JALXVM01	70.7	2.76	0	76	46617	89.3	2405	96.7	0
K. rhizophila p3-SID1455	JALXVE01	70.7	2.68	0	138	28174	88.9	2315	96.5	0
K. rhizophila p3-SID208	JALXRW01	70.9	2.61	0	120	32180	89.2	2247	96.9	0
K. rhizophila p3-SID284	JALXPY01	70.8	2.67	0	129	29728	89.1	2300	96.9	0
K. rhizophila p3-SID292	JALXPT01	70.8	2.63	0	120	37400	89.3	2300	96.2	0
K. rhizophila P7-4	AFID01	70.5	2.82	0	54	266116	88.6	2462	98.7	0
K. rhizophila RF	JPWX02	70.6	2.78	0	90	61655	88.6	2437	98.7	0
K. rhizophila SLM-01	JAQGES01	70.6	2.77	0	33	185515	88.7	2408	98.7	0
K. rhizophila TA68	RCOM01	70.7	2.70	0	195	25640	89.0	2363	98.0	0
K. rhizophila TNDT1	QMDP01000096	73.9	3.25	20	733	6041	88.6	3222	96.0	0.66
K. rhizophila TPW45	JWTC01	70.6	2.70	0	46	114097	88.7	2307	98.7	0
K. rhizophila UMB0131	PKJA01	70.5	2.83	121	8	2128678	88.1	2475	98.4	0
K. rhizophila UNH1	CP157319	70.5	2.80	0	1	2804693	88.3	2420	98.7	0
K. rhizophila ZSM2	JAUEPG010	70.8	2.65	0	31	202472	89.0	2296	99.3	0
K. rosea 13	CP127857	72.2	4.19	0	4	3815108	88.5	3801	99.3	0
K. rosea 640_1	CANLMJ01	72.1	4.30	0	47	374913	88.2	3940	99.3	0
K. rosea AF099C18	SLUD01	72.1	3.99	303	749	9873	88.5	4041	96.9	0
K. rosea ATCC 186	CP035103	72.8	3.95	0	1	3946651	88.9	3587	98.7	0

Strains	Accession No.	G+C content (%)	Genome size	Ns	Scaffolds	N50 (scaffolds)	Coding density (%)	Predicted genes	Complet-eness	Contami-nation
K. rosea ATCC 49321	QFBL01	72.5	4.02	0	59	283067	88.7	3716	99.3	0
K. rosea ATCC 516	QFBK01	72.8	3.86	0	91	145010	89.1	3547	99.3	0
K. rosea DE0293	VEKP01	72.1	4.21	326	281	219951	88.0	3990	99.3	0
K. rosea MSL 359	JAMAXV01	72.5	3.98	0	129	265844	88.6	3685	99.3	1.32
K. rosea NCTC7512	LR134487	72.9	3.81	0	1	3811253	89.2	3431	99.3	0
K. rosea NCTC7514	LR134391	72.9	3.76	0	1	3763037	89.2	3426	99.3	0
K. rosea NCTC7528	UGNO01	72.6	3.94	0	2	3791610	88.8	3549	99.3	0
K. rosea RP1	CP126483	72.4	3.96	0	4	3711540	88.6	3604	100.0	0
K. rosea S-A3	SMZT01	72.8	3.95	0	18	565760	89.4	3592	98.7	0
K. rosea SKFO21.2	JAXDEP01	72.2	4.19	0	303	33312	88.4	3953	99.3	0.55
K. rosea TA 28	JAMBOG01	73.1	3.64	0	73	158923	86.9	3373	99.3	0
K. rosea USHLN215	JBBCYR01	72.7	3.94	0	68	275426	88.9	3631	99.3	0
K. salina CV6	SSSF01	72.2	4.22	0	57	377329	88.4	3947	99.3	0
K. salsicia DE0195	VELM01	69.8	3.00	193	261	376152	87.8	2741	98.7	0
K. salsicia DE0232	VELE01	69.8	3.06	194	50	344584	88.7	2714	98.7	0
K. salsicia G1	CZJU01	70.4	3.00	0	199	29479	88.3	2672	98.7	3.29
K. salsicia JCM 16361	BAAARF01	70.2	2.78	10	26	252272	88.7	2455	98.7	0
K. sediminis JCM 17929	WOGU01	72.8	3.93	280	65	244575	87.4	3631	99.3	0
K. soli M5W7-7	RKMF01	67.0	2.95	0	31	187544	90.2	2644	99.3	0
Kocuria sp. 2-558A.fasta	CANMRA01	71.8	4.31	0	105	121406	87.6	3945	99.3	0
Kocuria sp. 27_S41	JAKDJD01	64.2	2.68	0	93	58842	89.5	2327	99.7	0
Kocuria sp. 2SI	SCNB01	70.2	2.97	0	58	134527	88.9	2612	99.3	0
Kocuria sp. 36	NOIS01	61.1	2.96	0	10	735507	89.3	2616	98.8	0
Kocuria sp. AG109	SNXQ01	71.9	2.37	192	15	337327	89.7	2070	98.3	0

Strains	Accession No.	G+C content (%)	Genome size	Ns	Scaffolds	N50 (scaffolds)	Coding density (%)	Predicted genes	Complet-eness	Contami-nation
Kocuria sp. APC 4018	JASDCY01	70.6	2.72	128	125	36183	88.5	2364	98.7	0
Kocuria sp. BT304	CP030039	71.2	2.76	0	1	2763150	87.9	2365	98.7	0
Kocuria sp. CCUG 69068	PDDL01	72.5	3.99	100	293	28714	88.9	3917	99.3	0.66
Kocuria sp. CTOTU49478	DAMCAF01	69.0	2.69	3460	98	51398	88.5	2485	98.0	0
Kocuria sp. CTOTU49686	DAMCFC01	69.0	2.71	10	25	175033	89.1	2436	98.0	0.33
Kocuria sp. CTOTU50499	DAMDIM01	68.8	2.85	5640	64	87211	88.7	2541	98.7	0
Kocuria sp. FE21-3	JAHWCN01	69.2	2.86	0	115	33425	89.5	2575	96.8	0.99
Kocuria sp. HMSC066H03	KV807502	70.5	2.77	1400	8	731430	88.4	2391	98.4	0
Kocuria sp. HSID16901	RBLM01	61.0	2.47	0	22	332879	90.3	2224	99.1	0
Kocuria sp. HSID17582	RBMA01	70.4	2.78	0	323	16287	88.2	2598	98.7	0
Kocuria sp. HSID17590	RBMB01	70.6	2.83	0	87	79906	88.4	2475	98.7	0
Kocuria sp. ICS0012	LZMM01	68.6	2.95	0	86	66873	88.2	2697	98.7	0.16
Kocuria sp. Im86wBCoDP_bin.76.MAG	CAMIAT01	71.5	2.46	0	99	50601	89.4	2159	97.8	0
Kocuria sp. JC486	JAAOZS01	67.0	2.86	511	61	108640	90.2	2539	99.3	0.66
Kocuria sp. KD4	CP050449	68.8	2.81	0	1	2806716	88.6	2498	99.3	0
Kocuria sp. KRD140	JABFAW01	68.9	2.74	6800	36	503376	87.8	2481	98.0	0
Kocuria sp. LUK	JAJMZI01	74.7	3.28	0	19	515231	89.3	2911	100.0	0
Kocuria sp. NBRC 114282	BSQO01	70.5	2.82	0	60	545959	88.7	2488	99.3	0
Kocuria sp. p3-SID1433	JALXVF01	70.3	2.74	0	165	24111	89.0	2436	97.9	0
Kocuria sp. PD6	JAUZUS01	68.8	2.88	0	40	251896	88.5	2621	99.3	0
Kocuria sp. PT10	UTAC01	70.7	2.65	0	40	131397	88.9	2299	98.7	0
Kocuria sp. S11A_Sa_4	JAUMTZ01	71.6	2.58	605	21	263080	90.3	2317	98.7	0.13
Kocuria sp. S27M_St_6	JAUNHD010	70.2	2.74	150	15	297108	89.0	2382	98.0	0
Kocuria sp. S28M_Sa_16	JAUNHG01	57.9	2.32	220	7	710470	91.5	2097	99.2	0

Strains	Accession No.	G+C content (%)	Genome size	Ns	Scaffolds	N50 (scaffolds)	Coding density (%)	Predicted genes	Complet-eness	Contami-nation
Kocuria sp. S36M_Sa_5	JAUNPE01	65.0	2.87	5380	349	15674	89.9	2783	97.4	2.63
Kocuria sp. SL71	JAPPRE01	70.3	2.89	0	2	2887229	87.4	2582	98.7	0
Kocuria sp. SM24M-10	LDNX01	72.3	4.35	0	177	45611	87.2	4148	99.3	0.77
Kocuria sp. TGY1120_3	AP022830	64.6	2.93	0	4	2838001	88.5	2558	99.7	0
Kocuria sp. TGY1127_2	AP022834	60.9	3.00	0	2	2963611	89.6	2661	98.8	0
Kocuria sp. tiJtcQB7Pi_bin.42.MAG	CALTUL01	61.0	2.44	0	58	64870	90.3	2195	99.5	0
Kocuria sp. UBA1838	DDIY01	68.9	2.69	16275	19	203777	87.5	2422	98.0	0
Kocuria sp. UBA5001	DGYR01	70.2	2.77	3487	21	253575	88.7	2443	96.4	0
Kocuria sp. UCD-OTCP	AOSQ01	72.7	3.79	190	68	101818	89.3	3464	99.3	0
Kocuria sp. WN036	NSKF01	72.7	3.89	0	77	120589	88.8	3599	98.7	0.33
Kocuria sp. WRN011	NSGI01	64.0	3.33	0	23	333879	89.6	3037	99.3	0
Kocuria sp. zatAbNZ7KQ bin.17.MAG	CAMIFL01	70.3	2.71	0	115	41625	89.2	2463	97.4	1.08
Kocuria sp. ZOR0020	JROX01	63.8	3.01	0	469	17949	89.0	2924	99.3	1.97
K. subflava YIM 13062	JAAVUN01	64.4	3.03	100	284	102588	89.7	2883	99.3	4.08
K. turfanensis HO-9042	CP014480	72.8	4.16	45	5	3742169	87.5	3830	98.7	0
K. tytonicola 473	RDEX01	71.1	2.64	0	5	1844606	89.1	2293	98.7	0
K. tytonicola DSM 104133	PHOA01000100	71.0	2.61	0	239	21669	88.7	2390	98.7	0
K. tytonis 442	PNJG02	70.8	2.72	0	9	746311	88.9	2396	98.7	0.33
K. varians 80	CP059343	70.5	2.82	0	1	2823038	88.2	2411	98.7	0
K. varians G6	CZJX01	70.5	2.90	0	95	104053	88.9	2588	99.3	0.66
K. varians NBRC 15358	BJNW01	70.6	2.84	0	48	111769	89.2	2480	99.3	0
M1N1S27	JBISWM01	72.8	3.65	0	60	147864	88.3	3339	99.3	0.22
M1R5S2	JBISWL01	72.4	4.00	0	155	46030	88.9	3624	99.3	1.32
M4R2S49	JBISWK01	72.0	4.20	0	190	36076	88.0	3839	99.3	2.63

Strains	Accession No.	G+C content (%)	Genome size	Ns	Scaffolds	N50 (scaffolds)	Coding density (%)	Predicted genes	Complet-eness	Contami-nation
M4R5S9	JBISWJ01	72.6	3.75	0	115	49104	87.9	3404	99.3	0
Rothia amarae JCM 11375	BAAAHX01	52.2	2.34	4	21	228729	88.7	2126	99.3	0
Rothia dentocariosa ATCC 17931	CP002280	53.7	2.51	0	1	2506025	85.5	2152	99.3	0

Table S2 Differentiating characteristics of the six strains with their closest relative.

Strains: **1,** strain M1R5S2^{T}**; 2,** strain M4R5S9^{T}**; 3,** strain M1N1S27**; 4,** strain M4R2S49^{T}**; 5,** strain HR5S1**; 6,** *K. rosea* CGMCC 4.7049^{T}

Notes: +, positive; -, negative; ND, no data.

**Characteristics**	**1**	**2**	**3**	**4**	**5**	**6**
Cell shape	Coccid	Coccid	Coccid	Coccid	Coccid	Coccid
Colony color	Pale yellow	Orange	Orange	Orange	Orange	Orange
Temperature range/optimal for growth (°C)	10-40(30-35)	10-40(30)	10-40(30)	10-40(30)	10-40(30)	ND
pH range/optimal for growth	6-11(8)	6-11(8)	6-11(8)	6-11(8)	6-11(8)	ND
NaCl range/optimal for growth (%, m/v)	0-20(0-3)	0-20(5)	0-20(5)	0-10(0)	0-10(0)	ND
Nitrate reduction	+	-	-	+	-	+
Gelatin hydrolysis	+	-	-	-	-	-
Alkaline phosphatase	-	-	-	+	-	+
Lipase(C14)	-	+	+	-	+	+
Cystine arylamidase	-	+	-	+	+	+

Trypsin	-	+	-	+	+	+
Acid phosphatase	-	-	+	-	+	+
β-Glucuronidase	+	+	+	-	-	-
β-Glucuronidase	+	+	+	-	-	+
N-Acetyl-β-glucosaminidase	-	-	-	-	+	-
α-Mannosidase	+	-	-	-	-	-
**Assimilation**						
D-Glucose	+	-	+	+	+	+
L-Arabinose	-	+	-	+	+	+
D-Mannose	+	+	+	+	-	+
N-Acetyl-glucosamine	+	-	-	-	-	-
D-Maltose	+	+	+	+	+	+
Adipic acid	+	+	-	+	+	+
Malic acid	+	+	-	+	+	+
Trisodium citrate	-	-	+	-	+	+
Phenylacetic acid	+	-	+	-	+	+
Genomic DNA G+C content (%)*	72.4	72.6	72.8	72.0	72.1	72.7

* The results were calculated according to whole genome sequences.

Table S3 Abbreviation of the name of the genes in Figure 3

**Abbreviations**	**Genes**
alkB	alkane 1-monoxygenae gene
benA	benzoate 1,2-dioxygenase large subunit gene
benB	benzoate 1,2-dioxygenase small subunit gene
benC	benzoate 1,2-dioxygenase electron transfer component
benD	dihydroxycyclohexa-2,4-diene-1-carboxylate dehydrogenase gene
benE	benzoate/H(+) symporter BenE family transporter gene
C23O	Catechol-2,3-dioxygenase gene
cadI	ArsI/CadI family heavy metal resistance metalloenzyme gene
carC	2-hydroxy-6-oxo-6-(2'-aminophenyl) hexa-2,4-dienoic acid hydrolase gene
carD	putative enoyl-CoA hydratase gene
catA	catechol 1,2-dioxygenase gene, partial
catC	muconolactone Delta-isomerase gene
catD	3-oxoadipate enol-lactonase gene
catD/pcaD	3-oxoadipate enol-lactonase gene
catI	oxoacid CoA-transferase subunit A gene
catJ	oxoacid CoA-transferase subunit B gene
clcA	catechol 1,2-dioxygenase/Chlorocatechol 1,2-dioxygenase gene
crt	enoyl-CoA hydratase/isomerase family protein gene
dodA	DOPA dioxygenase extradiol gene
echA8	enoyl-CoA hydratase/isomerase family protein gene
entH	PaaI family thioesterase gene
fabG	3-oxoacyl-ACP reductase gene
fabL	Enoyl-[acyl-carrier-protein] reductase [NADPH] gene
fadD	o-succinylbenzoate--CoA ligase gene
feaB	Phenylacetaldehyde dehydrogenase gene
fgd	LLM class flavin-dependent oxidoreductase gene
genK	**Gentisate transporter** gene
hapE	4-hydroxyacetophenone monooxygenase gene
hcaB	3-phenylpropionate-dihydrodiol/cinnamic acid-dihydrodiol dehydrogenase gene
hcaC	3-phenylpropionate/cinnamic acid dioxygenase ferredoxin subunit gene
hcaR	LysR family transcriptional regulator gene
hcaR2	Hca operon transcriptional activator HcaR gene
hcaR3	Hca operon transcriptional activator gene
hmgA	homogentisate 1,2-dioxygenase gene
hpaC3	4-hydroxyphenylacetate 3-monooxygenase, reductase component gene
hpaH	4-hydroxyphenylacetate 3-hydroxylase N-terminal domain-containing protein gene
hpcG	2-oxo-hept-4-ene-1,7-dioate hydratase gene
hpcH	4-hydroxy-2-oxo-heptane-1,7-dioate aldolase gene

hpcH/hpaI	HpcH/HpaI aldolase/citrate lyase family protein gene
linX	2,5-dichloro-2,5-cyclohexadiene-1,4-diol dehydrogenase LinX gene
mdlC	benzoylformate decarboxylase gene
menA	1,4-dihydroxy-2-naphthoate polyprenyltransferase gene
menD	succinyl-5-enolpyruvyl-6-hydroxy-3-cyclohexene-1-carboxylic-acid synthase gene
menE	2-succinylbenzoate--CoA ligase gene
menE2	putative 2-succinylbenzoate--CoA ligase gene
menH	2-succinyl-6-hydroxy-2,4-cyclohexadiene-1-carboxylate synthase gene
mhbT	3-hydroxybenzoate transporter gene
mhpA	3-(3-hydroxy-phenyl) propionate/3-hydroxycinnamic acid hydroxylase gene
mobA	3-hydroxybenzoate 4-monooxygenase gene
oleD	2-alkyl-3-oxoalkanoate reductase gene
paaA	phenylacetyl-CoA epoxidase subunit gene
paaB	1,2-phenylacetyl-CoA epoxidase subunit gene
paaC	phenylacetyl-CoA epoxidase subunit gene
paaD	phenylacetyl-CoA epoxidase subunit gene
paaE	phenylacetyl-CoA epoxidase subunit gene
paaI	PaaI family thioesterase gene
paaI/entH	PaaI family thioesterase gene
paaJ	3-oxoadipyl-CoA/3-oxo-5,6-dehydrosuberyl-CoA thiolase gene
paaK	phenylacetate--CoA ligase family protein gene
pcaB	3-carboxy-cis, cis-muconate cycloisomerase gene
pcaC	carboxymuconolactone decarboxylase gene
pcaD	3-oxoadipate enol-lactonase gene
pcaF	thiolase family protein gene
pcaG	protocatechuate 3,4-dioxygenase subunit alpha gene
pcaH	protocatechuate 3,4-dioxygenase subunit beta gene
pcaI	3-oxoacid CoA-transferase subunit A gene
pcaJ	oxoacid CoA-transferase subunit A gene
pcaK	4-hydroxybenzoate transporter gene
pcaR	IclR family transcriptional regulator C-terminal domain-containing protein gene
phzF	Trans-2,3-dihydro-3-hydroxyanthranilate isomerase gene
pikA2	Narbonolide/10-deoxymethynolide synthase gene
pobA	3-hydroxybenzoate 4-monooxygenase gene
pobB	Phenoxybenzoate dioxygenase subunit B gene
ppo	polyphenol oxidase family protein gene
pptA	tautomerase family protein gene
praI	4-hydroxybenzoate 3-monooxygenase gene
rpf	transglycosylase family protein gene
sdgD	Gentisate 1,2-dioxygenase gene
SDR	short chain dehydrogenase gene

sotB	MFS transporter gene
tfdB	2,4-dichlorophenol 6-monooxygenase gene
todF	2-hydroxy-6-oxo-2,4-heptadienoate hydrolase gene
tsaM	aromatic ring-hydroxylating dioxygenase subunit alpha gene
tyrA	prephenate dehydrogenase gene
vanA	Vanillate/3-O-methylgallate O-demethylase gene
vanB	PDR/VanB family oxidoreductase gene
vanB/pobB	PDR/VanB family oxidoreductase/Phenoxybenzoate dioxygenase subunit beta gene
vdh	Vanillin dehydrogenase gene
xlnD	3-hydroxybenzoate 6-hydroxylase gene
xylC	Benzaldehyde dehydrogenase gene
xylD	dihydroxy-acid dehydratase gene
ydhP	Inner membrane transport protein YdhP gene
yeaX	MFS transporter, partial gene
ygiD	DOPA dioxygenase extradiol gene
yhjE	Inner membrane metabolite transport protein gene

Fig.S1 Comparative genomic analysis results

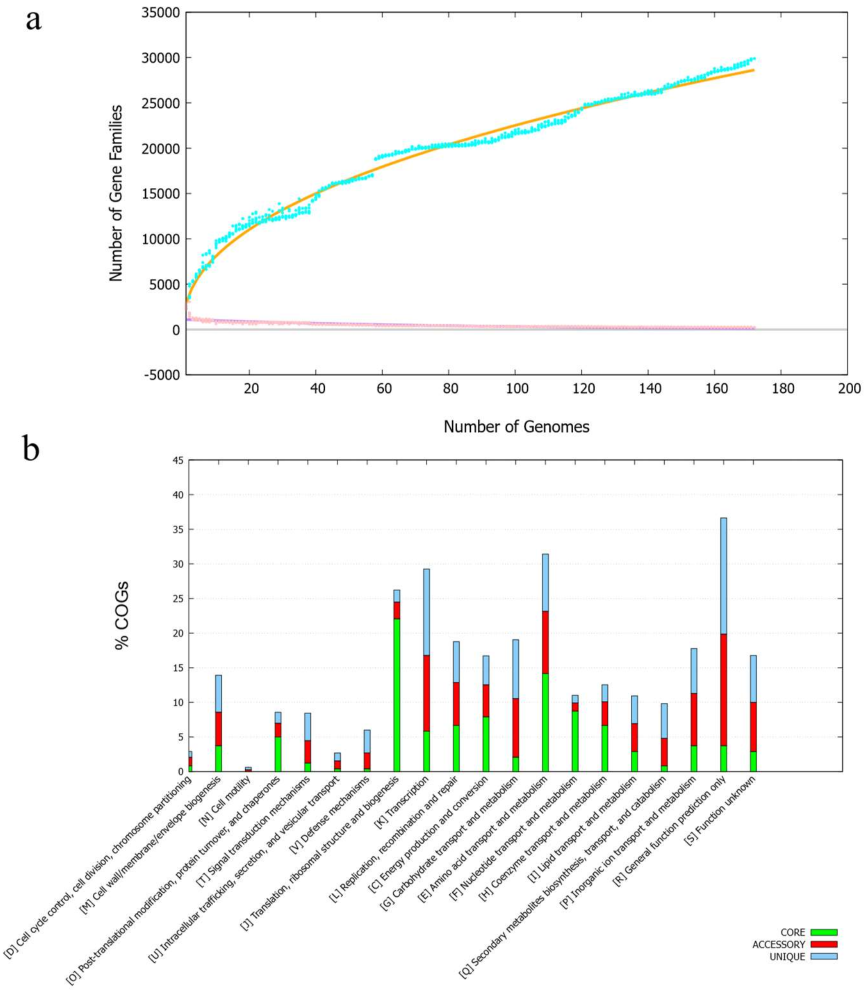

Fig. S2 Prediction of the central metabolic potential of *Kocuria* strains, _

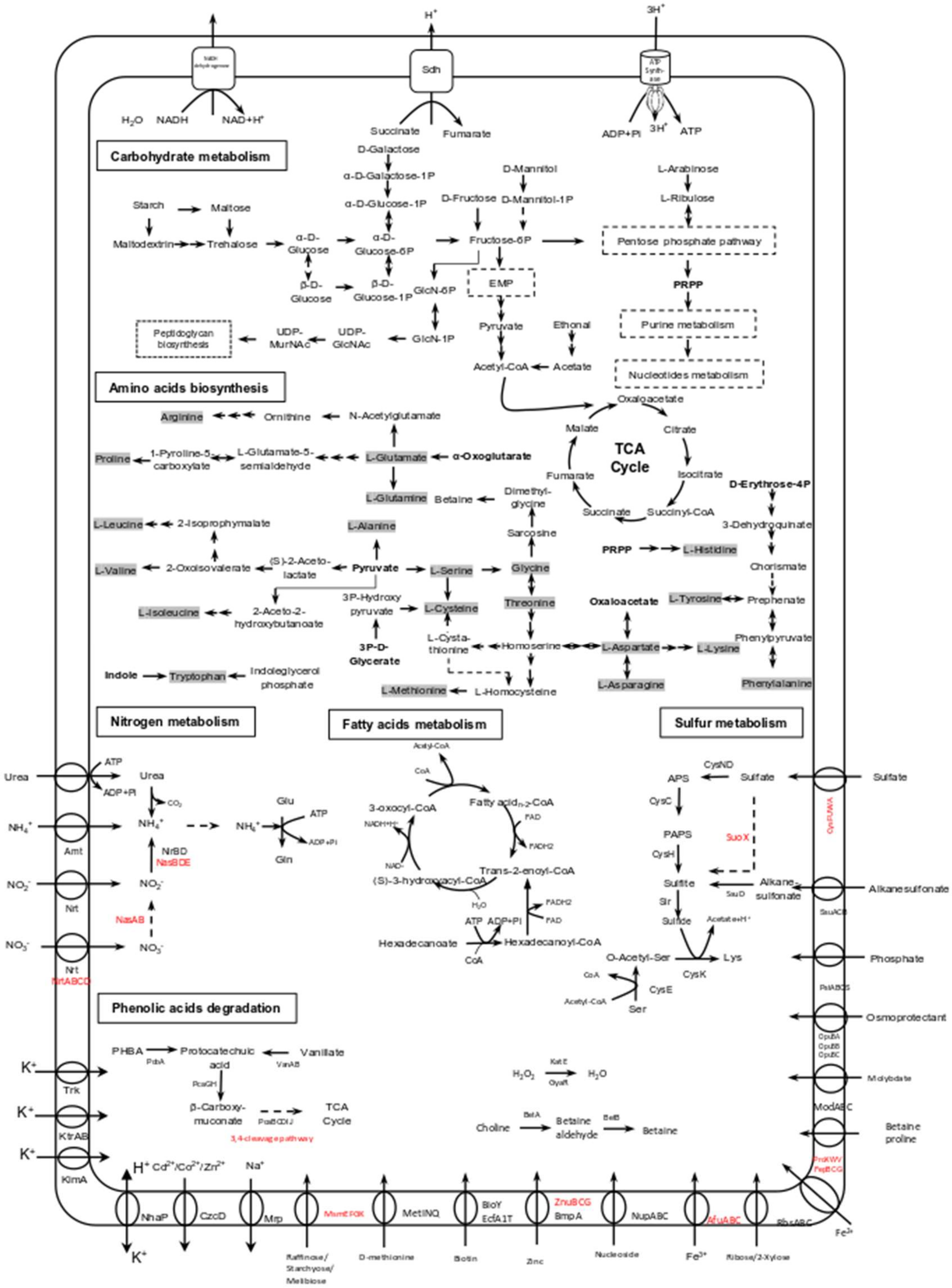

Supplement: Supplementary file 1 [file Presentation_1.pdf]
